# Supplementary material for: Evolutionary dynamics of mycorrhizal symbiosis in land plant diversification
Source: Sci Rep. 2018 Jul 16;8:10698. doi: 10.1038/s41598-018-28920-x (PMC6048063; doi:10.1038/s41598-018-28920-x)
Supplement: Supplementary file 1 — Supplementary Information [file 41598_2018_28920_MOESM1_ESM.pdf]

Supplementary information for

Evolutionary dynamics of mycorrhizal symbiosis in land plant diversification

Authors

Frida A.A. Feijen, Rutger A. Vos, Jorinde Nuytinck & Vincent S.F.T. Merckx

Supplementary Figures

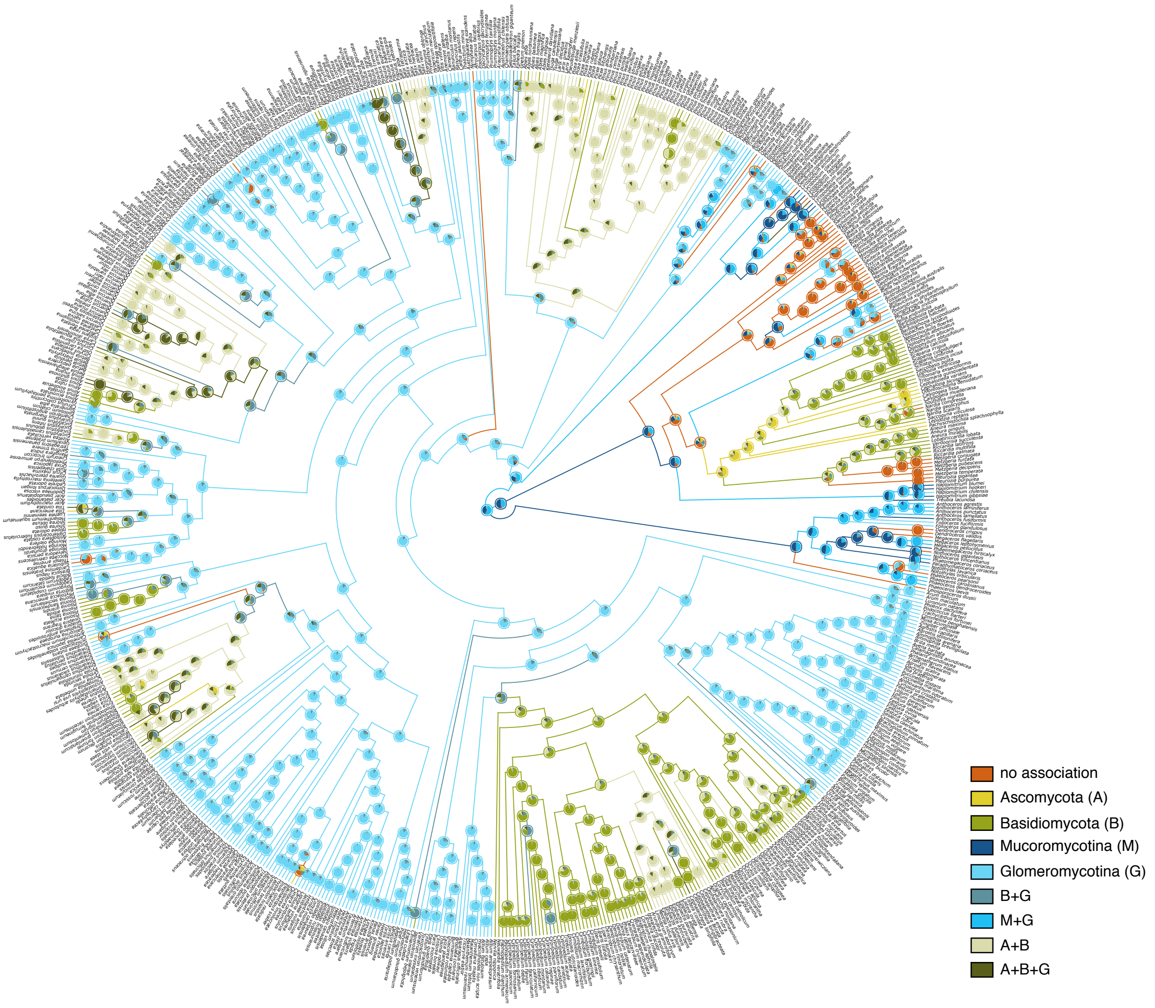

Figure S1 | Ancestral state reconstruction of mycorrhizal associations in land plants. Cladogram showing the ancestral state reconstructions of mycorrhizal associations in land plants (n = 732 species) using a phylogenetic hypothesis in which a clade consisting of liverworts and bryophytes are the sister group of all other land plant species. Branches are coloured according to the most probable state of their ancestral nodes. Pie charts show the likelihood of the ancestral states for each node.

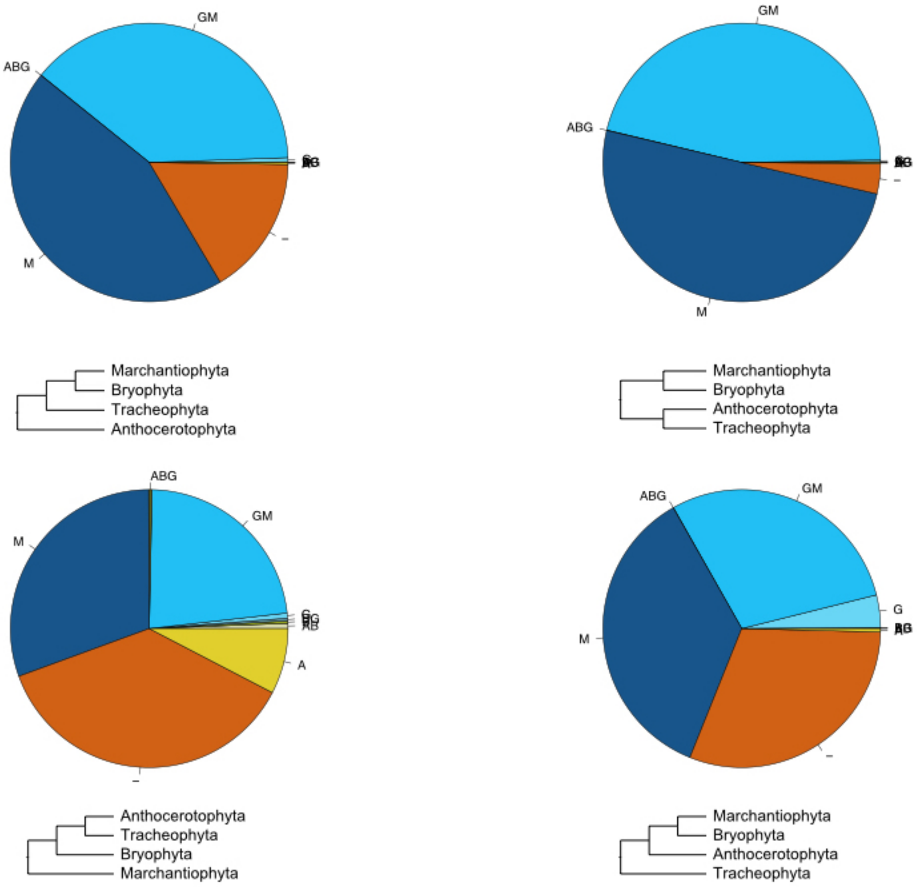

Figure S2 | Ancestral state reconstruction of the mycorrhizal association for the last common ancestor of Embryophyta, given the different phylogenetic constraints. Pie charts show the likelihood of the ancestral states for the MRCA of Embryophyta for each phylogenetic hypothesis shown below. Letters represent mycorrhizal associations: (A) Ascomycota; (B) Basidiomycota; (G) Glomeromycotina; (M) Mucoromycotina; (-) Non-mycorrhizal. Combinations of letters represent a combination of mycorrhizal associations.

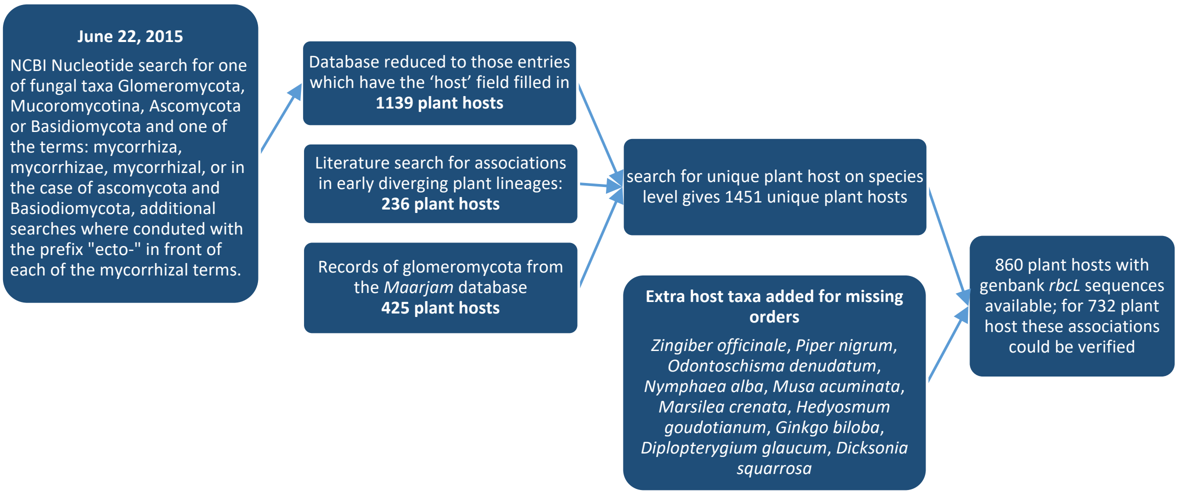

Figure S3 | PRISMA flow diagram depicting the flow of records through the different phases of the database construction. A database was constructed using NCBI Nucleotide records of potential mycorrhizal fungi and their hosts. Separate literature searches where conducted for liverworts, hornworts, ferns and lycopods and plant orders that where still missing from the database. The resulting set included 860 plant hosts for which *rbcL* sequences were available for reconstructing the plant host phylogeny. Mycorrhizal status for these 860 records was verified with literature searches and records where discarded when these could not be verified. The final dataset includes 732 plant species distributed over 78 plant orders.

Supplementary Tables

Table S1 | An enumeration of the most likely scenarios by which the initial association between mycorrhiza and land plants came about, with their relative support by the data quantified.

Letters represent mycorrhizal associations: (A) Ascomycota; (B) Basidiomycota; (G) Glomeromycotina; (M) Mucoromycotina; (-) Non-mycorrhizal.

Rooting	Constrained root state	lnL-run1	lnL-run2	lnL-run3	MeanLnL	Log Bayes Factor
**ATxMB**	**M**	-679.911621	-676.876677	-671.750591	-676.1796297	**30.970172**
**ATxMB**	**A**	-667.689857	-662.379807	-660.662247	-663.5773037	5.76552
**ATxMB**	**B**	-659.387552	-654.114889	-655.512716	-656.3383857	-8.712316
**ATxMB**	**G**	-658.944728	-664.071205	-658.896524	-660.6374857	-0.114116
**ATxMB**	**none**	-657.828967	-663.990698	-660.263966	-660.6945437	
**ABasal**	**M**	-659.531851	-660.907574	-661.371545	-660.6036567	**8.352606**
**ABasal**	**A**	-658.200433	-655.505655	-659.652527	-657.786205	2.717702667
**ABasal**	**B**	-652.784653	-655.987546	-652.970134	-653.914111	-5.026485333
**ABasal**	**G**	-660.683007	-657.611251	-655.600053	-657.9647703	3.074833333
**ABasal**	**none**	-656.260611	-656.820943	-656.200507	-656.4273537	
**MBasal**	**M**	-638.636828	-638.362954	-639.758555	-638.9194457	**13.25466133**
**MBasal**	**A**	-630.678902	-632.050728	-631.148102	-631.2925773	-1.999075333
**MBasal**	**B**	-629.176058	-631.08574	-630.670126	-630.3106413	-3.962947333
**MBasal**	**G**	-633.478991	-633.849004	-634.833786	-634.053927	3.523624
**MBasal**	**none**	-632.515668	-631.542916	-632.817761	-632.292115	
**TBasal**	**M**	-677.357096	-665.456265	-667.230832	-670.014731	**13.26064867**
**TBasal**	**A**	-658.521647	-676.24561	-667.693472	-667.4869097	8.205006
**TBasal**	**B**	-663.959132	-656.572985	-660.663275	-660.398464	-5.971885333
**TBasal**	**G**	-660.874689	-665.269231	-658.571373	-661.5717643	-3.625284667
**TBasal**	**none**	-665.074366	-670.923572	-654.155282	-663.3844067	

Table S2 | Mycorrhizal interactions database

order	genus	species	Synonym/subspecies/alternative spelling	Ascomycota	Basidiomycota	Glomeromycota	Mucoromycotina	References
**Anthocerotophyta**								
Anthocerotales	*Anthoceros*	*agrestis*		0	0	1	1	[1]
Anthocerotales	*Anthoceros*	*fusiformis*		0	0	0	1	[1]
Anthocerotales	*Anthoceros*	*lamellatus*		0	0	1	1	[1]
Anthocerotales	*Anthoceros*	*laminiferus*		0	0	1	1	[1,2]
Anthocerotales	*Anthoceros*	*punctatus*		0	0	1	1	[1]
Anthocerotales	*Folioceros*	*fuciformis*		0	0	1	0	[1]
Anthocerotales	*Folioceros*	*glandulosus*	Folioceros cf. glandulosus	0	0	1	0	[1]
Dendrocerotales	*Dendroceros*	*crispus*		0	0	0	0	[1]
Dendrocerotales	*Dendroceros*	*validus*		0	0	0	0	[1]
Dendrocerotales	*Megaceros*	*flagellaris*		0	0	0	0	[1]
Dendrocerotales	*Megaceros*	*leptohymenius*		0	0	1	1	[1]
Dendrocerotales	*Megaceros*	*pellucidus*		0	0	1	1	[1]
Dendrocerotales	*Nothoceros*	*giganteus*		0	0	0	0	[1]
Dendrocerotales	*Nothoceros*	*vincentianus*		0	0	1	1	[1]
Dendrocerotales	*Phaeomegaceros*	*coriaceus*	Phaeomegaceros coriaceus[3,4]	0	0	1	1	[1]
Dendrocerotales	*Phaeomegaceros*	*hirticalyx*		0	0	0	1	[1]
Leiosporocerotales	*Leiosporoceros*	*dussii*		0	0	0	0	[1]
Notothyladales	*Notothylas*	*javanica*		0	0	1	0	[1]
Notothyladales	*Notothylas*	*orbicularis*		0	0	1	0	[1]
Notothyladales	*Paraphymatoceros*	*coriaceus*		0	0	0	1	[2]
Notothyladales	*Phaeoceros*	*carolinianus*		0	0	1	1	[1,2]
Notothyladales	*Phaeoceros*	*dendroceroides*		0	0	1	1	[1]
Notothyladales	*Phaeoceros*	*laevis*		0	0	1	1	[1,2]
Notothyladales	*Phaeoceros*	*pearsonii*		0	0	0	0	[1]
**Bryophyta**								
Funariales	*Physcomitrella*	*patens*		0	0	0	0	[5]
Hypnales	*Sanionia*	*uncinata*		0	0	0	0	[5]
Orthotrichales	*Nyholmiella*	*obtusifolia*		0	0	0	0	[5]
Pottiales	*Syntrichia*	*ruralis*		0	0	0	0	[5]
Sphagnales	*Sphagnum*	*palustre*		0	0	0	0	[5]
Takakiales	*Takakia*	*lepidozioides*		0	0	0	0	[5]
Tetraphidales	*Tetraphis*	*pellucida*		0	0	0	0	[5]
**Marchantiophyta**								
Blasiales	*Blasia*	*pusilla*		0	0	0	0	[6]
Calobryales	*Haplomitrium*	*blumei*		0	0	0	1	[2]
Calobryales	*Haplomitrium*	*chilensis*	Haplomitrium chiliensis	0	0	1	0	[6,7]
Calobryales	*Haplomitrium*	*gibbsiae*		0	0	0	1	[2]
Calobryales	*Haplomitrium*	*hookeri*		0	0	0	1	[2]
Fossombroniales	*Allisonia*	*cockaynii*		0	0	1	1	[2]
Fossombroniales	*Austrofossombronia*	*australis*		0	0	1	0	[2,7]
Fossombroniales	*Fossombronia*	*angulosa*		0	0	1	0	[7]
Jungermanniales	*Barbilophozia*	*barbata*		0	1	0	0	[8]
Jungermanniales	*Barbilophozia*	*hatcheri*		0	1	0	0	[8]
Jungermanniales	*Barbilophozia*	*lycopodioides*		0	1	0	0	[8]
Jungermanniales	*Calypogeia*	*fissa*		1	0	0	0	[8-10]
Jungermanniales	*Calypogeia*	*muelleriana*		1	1	0	0	[5,8,10,11]
Jungermanniales	*Cephalozia*	*bicuspidata*		1	0	0	0	[9,10]
Jungermanniales	*Cephaloziella*	*varians*		1	0	0	0	[12]
Jungermanniales	*Diplophyllum*	*albicans*		0	1	0	0	[8]
Jungermanniales	*Diplophyllum*	*obtusifolium*		0	1	0	0	[8]
Jungermanniales	*Isopaches*	*bicrenatus*	Lophozia bicrenata[13]	0	1	0	0	[8]
Jungermanniales	*Lepidozia*	*reptans*		1	0	0	0	[8-10]
Jungermanniales	*Lophozia*	*ventricosa*		0	1	0	0	[8-10]
Jungermanniales	*Nardia*	*compressa*		0	0	0	0	[14]
Jungermanniales	*Nardia*	*geoscyphus*		0	1	0	0	[8]
Jungermanniales	*Nardia*	*scalaris*		0	1	0	0	[8-10]
Jungermanniales	*Neoorthocaulis*	*floerkei*	Barbilophozia floerckii[15]	0	1	0	0	[8]
Jungermanniales	*Odontoschisma*	*denudatum*		1	0	0	0	[9,10]
Jungermanniales	*Orthocaulis*	*attenuatus*	Barbilophozia attenuata[16]	0	1	0	0	[8,14]
Jungermanniales	*Pachyschistochila*	*splachnophylla*		1	0	0	0	[9,17]
Jungermanniales	*Saccobasis*	*polita*	Tritomaria polita [18]	0	1	0	0	[8]
Jungermanniales	*Saccogyna*	*viticulosa*		0	1	0	0	[8,10]
Jungermanniales	*Scapania*	*calcicola*		0	1	0	0	[8]
Jungermanniales	*Scapania*	*cuspiduligera*		0	1	0	0	[8]
Jungermanniales	*Scapania*	*irrigua*		0	1	0	0	[8]
Jungermanniales	*Scapania*	*umbrosa*		0	1	0	0	[8]
Jungermanniales	*Schistochilopsis*	*incisa*	Lophozia incisa [19]	0	1	0	0	[11]
Jungermanniales	*Southbya*	*nigrella*		0	1	0	0	[8]
Jungermanniales	*Tritomaria*	*exsectiformis*		0	1	0	0	[8]
Jungermanniales	*Tritomaria*	*quinquedentata*		0	1	0	0	[8,10]
Marchantiales	*Bucegia*	*romanica*		0	0	0	0	[6]
Marchantiales	*Conocephalum*	*conicum*		0	0	1	0	[6,7]
Marchantiales	*Exormotheca*	*pustulosa*		0	0	0	0	[6]
Marchantiales	*Mannia*	*androgyna*	Mannia angrogyna	0	0	0	0	[6]
Marchantiales	*Mannia*	*fragrans*		0	0	0	0	[6]
Marchantiales	*Marchantia*	*paleacea*		0	0	1	0	[5,6]
Marchantiales	*Monoclea*	*gottschei*		0	0	1	0	[6,7]
Marchantiales	*Monosolenium*	*tenerum*		0	0	0	0	[6]
Marchantiales	*Oxymitra*	*incrassata*		0	0	0	0	[6]
Marchantiales	*Preissia*	*quadrata*		0	0	1	0	[7]

order	genus	species	Synonym/subspecies/alternative spelling	Ascomycota	Basidiomycota	Glomeromycota	Mucoromycotina	References
Marchantiales	*Riccia*	*huebeneriana*	Ricciella huebeneriana[20]	0	0	0	0	6
Marchantiales	*Sauteria*	*alpina*		0	0	0	0	6
Marchantiales	*Wiesnerella*	*denudata*		0	0	0	0	6
Metzgeriales	*Aneura*	*maxima*		0	1	0	0	8
Metzgeriales	*Aneura*	*mirabilis*	Cryptothallus mirabilis [21]	0	1	0	0	8,10
Metzgeriales	*Aneura*	*pinguis*		0	1	0	0	8,10,11,22
Metzgeriales	*Lobatiriccardia*	*lobata*	Aneura lobata [23]	0	1	0	0	6
Metzgeriales	*Metzgeria*	*conjugata*		0	0	0	0	6
Metzgeriales	*Metzgeria*	*decipiens*		0	0	0	0	6
Metzgeriales	*Metzgeria*	*furcata*		0	0	0	0	6
Metzgeriales	*Metzgeria*	*pubescens*	Apometzgeria pubescens [24]	0	0	0	0	6
Metzgeriales	*Metzgeria*	*temperata*		0	0	0	0	6
Metzgeriales	*Riccardia*	*latifrons*		0	1	0	0	22
Metzgeriales	*Riccardia*	*multifida*		0	1	0	0	22
Metzgeriales	*Riccardia*	*palmata*		0	1	0	0	22
Metzgeriales	*Verdoornia*	*succulenta*		0	1	0	0	5,25
Neohodgsoniales	*Neohodgsonia*	*mirabilis*		0	0	1	1	2
Pallaviciniales	*Jensenia*	*connivens*		0	0	1	0	2,7
Pallaviciniales	*Pallavicinia*	*xiphoides*		0	0	0	0	6
Pallaviciniales	*Phyllothallia*	*nivicola*		0	0	0	0	6
Pallaviciniales	*Podomitrium*	*phyllanthus*		0	0	1	0	2,7
Pallaviciniales	*Symphyogyna*	*hymenophyllum*	Symphyogyna hymenophyton	0	0	1	0	2,7
Pelliales	*Pellia*	*endiviifolia*		0	0	1	0	6,7
Pleuroziales	*Pleurozia*	*gigantea*		0	0	0	0	6
Pleuroziales	*Pleurozia*	*purpurea*		0	0	0	0	6
Sphaerocarpales	*Geothallus*	*tuberosus*	Geothallus tuberosa	0	0	0	0	6
Sphaerocarpales	*Riella*	*helicophylla*		0	0	0	0	6
Sphaerocarpales	*Sphaerocarpos*	*texanus*		0	0	0	0	6
Treubiales	*Treubia*	*lacunosa*		0	0	0	1	2
**Tracheophyta, Angiosperm**								
Alismatales	*Arum*	*italicum*		0	0	1	0	7
Alismatales	*Arum*	*maculatum*		0	0	1	0	7
Alismatales	*Luronium*	*natans*		0	0	1	0	7
Apiales	*Aegopodium*	*podagraria*		0	0	1	0	7
Apiales	*Daucus*	*carota*		0	0	1	0	26
Apiales	*Hedera*	*rhombea*		0	0	1	0	27
Apiales	*Panax*	*ginseng*		0	0	1	0	28
Apiales	*Panax*	*japonicus*		0	0	1	0	7
Apiales	*Torilis*	*arvensis*		0	0	1	0	7
Apiales	*Torilis*	*japonica*		0	0	1	0	7
Arecales	*Phoenix*	*dactylifera*		0	0	1	0	7
Arecales	*Podococcus*	*barteri*		0	0	1	0	7
Arecales	*Trachycarpus*	*fortunei*		0	0	1	0	7
Asparagales	*Acianthus*	*exsertus*		0	1	0	0	29
Asparagales	*Allium*	*ampeloprasum*		0	0	1	0	30
Asparagales	*Allium*	*cepa*		0	0	1	0	7
Asparagales	*Allium*	*fistulosum*		0	0	1	0	31
Asparagales	*Anacamptis*	*laxiflora*		0	1	0	0	32
Asparagales	*Anacamptis*	*morio*		1	1	0	0	33
Asparagales	*Aphyllorchis*	*montana*		1	1	0	0	34
Asparagales	*Arundina*	*graminifolia*		0	1	0	0	35
Asparagales	*Asparagus*	*officinalis*		0	0	1	0	36,37
Asparagales	*Brodiaea*	*coronaria*		0	0	1	0	38
Asparagales	*Cephalanthera*	*damasonium*		1	1	0	0	39-42
Asparagales	*Cephalanthera*	*erecta*		0	1	0	0	39
Asparagales	*Cephalanthera*	*falcata*		0	1	0	0	39
Asparagales	*Cephalanthera*	*longibracteata*		0	1	0	0	39
Asparagales	*Cephalanthera*	*longifolia*		1	1	1	0	39,41,43
Asparagales	*Cephalanthera*	*rubra*		1	1	0	0	39,42
Asparagales	*Convallaria*	*majalis*		0	0	1	0	44
Asparagales	*Corallorhiza*	*trifida*		0	1	0	0	45
Asparagales	*Corycium*	*carnosum*		0	1	0	0	46
Asparagales	*Cremastra*	*appendiculata*		0	1	0	0	47
Asparagales	*Cymbidium*	*ensifolium*		0	1	0	0	48,49
Asparagales	*Cymbidium*	*floribundum*		0	1	0	0	50
Asparagales	*Cymbidium*	*goeringii*		0	1	0	0	51,52
Asparagales	*Cymbidium*	*lancifolium*		0	1	0	0	52
Asparagales	*Cymbidium*	*macrorhizon*		0	1	0	0	52
Asparagales	*Cymbidium*	*sinense*		0	1	0	0	51
Asparagales	*Cypripedium*	*acaule*		0	1	0	0	53
Asparagales	*Cypripedium*	*arietinum*		0	1	0	0	53
Asparagales	*Cypripedium*	*calceolus*		0	1	0	0	53,54
Asparagales	*Cypripedium*	*californicum*		0	1	1	0	53,54
Asparagales	*Cypripedium*	*candidum*		0	1	0	0	53,54
Asparagales	*Cypripedium*	*debile*		0	1	0	0	53
Asparagales	*Cypripedium*	*fasciculatum*		0	1	0	0	53,54
Asparagales	*Cypripedium*	*flavum*		0	1	0	0	55
Asparagales	*Cypripedium*	*formosanum*		0	1	0	0	53
Asparagales	*Cypripedium*	*guttatum*		0	1	0	0	53-55
Asparagales	*Cypripedium*	*japonicum*		0	1	0	0	53
Asparagales	*Cypripedium*	*montanum*		0	1	0	0	53,54
Asparagales	*Cypripedium*	*parviflorum*		0	1	1	0	53,54
Asparagales	*Cypripedium*	*reginae*		0	1	0	0	53
Asparagales	*Cypripedium*	*tibeticum*		0	1	0	0	55
Asparagales	*Dendrobium*	*catenatum*	Dendrobium officinale [56]	0	1	0	0	57,58
Asparagales	*Dendrobium*	*chrysanthum*		0	1	0	0	59

order	genus	species	Synonym/subspecies/alternative spelling	Ascomycota	Basidiomycota	Glomeromycota	Mucoromycotina	References
Asparagales	*Dendrobium*	*crumenatum*		0	1	0	0	[35]
Asparagales	*Dendrobium*	*fimbriatum*		0	1	0	0	[58]
Asparagales	*Dendrobium*	*nobile*		0	1	0	0	[59]
Asparagales	*Disa*	*bracteata*		0	1	0	0	[60,61]
Asparagales	*Disperis*	*capensis*		0	1	0	0	[46]
Asparagales	*Epidendrum*	*rhopalostele*		0	1	0	0	[62]
Asparagales	*Epipactis*	*albensis*		1	1	0	0	[63]
Asparagales	*Epipactis*	*atrorubens*		1	1	0	0	[41,42,63]
Asparagales	*Epipactis*	*dunensis*		1	1	0	0	[41]
Asparagales	*Epipactis*	*helleborine*		1	1	0	0	[42,63,64]
Asparagales	*Epipactis*	*palustris*		1	1	0	0	[42,65]
Asparagales	*Epipactis*	*purpurata*		1	1	0	0	[63]
Asparagales	*Eriochilus*	*cucullatus*		0	1	0	0	[64]
Asparagales	*Goodyera*	*foliosa*		1	1	0	0	[66]
Asparagales	*Goodyera*	*maximowicziana*	foliosa var. maximowicziana.	0	1	0	0	[66]
Asparagales	*Goodyera*	*oblongifolia*		0	1	0	0	[54]
Asparagales	*Goodyera*	*procera*		1	1	0	0	[66]
Asparagales	*Goodyera*	*pubescens*		0	1	0	0	[67]
Asparagales	*Goodyera*	*repens*		0	1	0	0	[66]
Asparagales	*Goodyera*	*schlechtendaliana*		0	1	0	0	[66]
Asparagales	*Goodyera*	*velutina*		1	1	0	0	[66]
Asparagales	*Gymnadenia*	*conopsea*		1	1	0	0	[65,68,69]
Asparagales	*Hexalectris*	*revoluta*		0	1	0	0	[64]
Asparagales	*Hyacinthoides*	*non-scripta*		0	0	1	0	[7]
Asparagales	*Ionopsis*	*utricularioides*		0	1	0	0	[70]
Asparagales	*Limodorum*	*abortivum*		1	1	0	0	[71]
Asparagales	*Liparis*	*kumokiri*		0	1	0	0	[72]
Asparagales	*Liparis*	*liliifolia*	Liparis lilifolia	0	1	0	0	[67]
Asparagales	*Liparis*	*loeselii*		0	1	0	0	[65]
Asparagales	*Listera*	*cordata*	Neottia cordata[73]	0	1	0	0	[74]
Asparagales	*Listera*	*ovata*	Neottia ovata[73]	0	1	0	0	[74]
Asparagales	*Maianthemum*	*bifolium*		0	0	1	0	[7]
Asparagales	*Maianthemum*	*racemosum*		0	0	1	0	[7]
Asparagales	*Neottia*	*nidus-avis*		1	1	0	0	[42,64,74,75]
Asparagales	*Nervilia*	*nipponica*		0	1	0	0	[76]
Asparagales	*Neuwiedia*	*veratrifolia*		0	1	0	0	[53]
Asparagales	*Ophrys*	*fuciflora*		0	1	0	0	[32]
Asparagales	*Ophrys*	*insectifera*		0	1	0	0	[77]
Asparagales	*Orchis*	*anthropophora*		0	1	0	0	[78,79]
Asparagales	*Orchis*	*mascula*		0	1	0	0	[79]
Asparagales	*Orchis*	*militaris*		0	1	0	0	[79]
Asparagales	*Orchis*	*purpurea*		0	1	0	0	[79]
Asparagales	*Orchis*	*simia*		0	1	0	0	[78]
Asparagales	*Paphiopedilum*	*armeniacum*		0	1	0	0	[55]
Asparagales	*Paphiopedilum*	*dianthum*		0	1	0	0	[55]
Asparagales	*Phaius*	*tancarvilleae*	Phaius tankervilliae	0	1	0	0	[80,81]
Asparagales	*Platanthera*	*azorica*		0	1	0	0	[82]
Asparagales	*Platanthera*	*chlorantha*		1	1	0	0	[42]
Asparagales	*Platanthera*	*micrantha*		0	1	0	0	[82]
Asparagales	*Pseudorchis*	*albida*		1	1	0	0	[83]
Asparagales	*Pterostylis*	*nutans*		0	1	0	0	[84]
Asparagales	*Pterygodium*	*catholicum*		0	1	0	0	[46]
Asparagales	*Pyrorchis*	*nigricans*		0	1	0	0	[61]
Asparagales	*Rhomboda*	*cristata*	Hetaeria cristata[85]	0	1	0	0	[66]
Asparagales	*Serapias*	*cordigera*		0	1	0	0	[86]
Asparagales	*Spiranthes*	*sinensis*		0	1	0	0	[72]
Asparagales	*Tainia*	*latifolia*		0	1	0	0	[49]
Asparagales	*Tipularia*	*discolor*		0	1	0	0	[67]
Asparagales	*Tricoryne*	*elatior*		0	0	1	0	[7]
Asparagales	*Trizeuxis*	*falcata*		0	1	0	0	[87]
Asparagales	*Wullschlaegelia*	*aphylla*		0	1	0	0	[88]
Asparagales	*Zeuxine*	*agyokuana*	Hetaeria agyokuana	0	1	0	0	[66]
Asparagales	*Zeuxine*	*strateumatica*		0	1	0	0	[49]
Asterales	*Achillea*	*millefolium*		0	0	1	0	[7]
Asterales	*Antennaria*	*dioica*		0	0	1	0	[7]
Asterales	*Arnica*	*montana*		0	0	1	0	[7]
Asterales	*Artemisia*	*frigida*		0	0	1	0	[7]
Asterales	*Artemisia*	*ludoviciana*		0	0	1	0	[7]
Asterales	*Artemisia*	*vulgaris*		0	0	1	0	[7]
Asterales	*Aster*	*tripolium*		0	0	1	0	[7]
Asterales	*Campanula*	*punctata*		0	0	1	0	[7]
Asterales	*Carduus*	*tenuiflorus*		0	0	1	0	[7]
Asterales	*Centaurea*	*jacea*		0	0	1	0	[7]
Asterales	*Conyza*	*canadensis*		0	0	1	0	[89]
Asterales	*Cynara*	*cardunculus*	Cynara scolymus	0	0	1	0	[7]
Asterales	*Elephantopus*	*scaber*		0	0	1	0	[7]
Asterales	*Helianthus*	*annuus*		0	0	1	0	[7]
Asterales	*Helichrysum*	*stoechas*		0	0	1	0	[90]
Asterales	*Heterotheca*	*villosa*		0	0	1	0	[7]
Asterales	*Hypochaeris*	*radicata*	Hypochoeris radicata	0	0	1	0	[7]
Asterales	*Inula*	*conyza*		0	0	1	0	[91,92]
Asterales	*Jacobaea*	*vulgaris*		0	0	1	0	[7]
Asterales	*Leontodon*	*hispidus*		0	0	1	0	[93]
Asterales	*Ligularia*	*virgaurea*		0	0	1	0	[7]
Asterales	*Lobelia*	*dortmanna*		1	0	1	0	[7,94]
Asterales	*Pilosella*	*officinarum*	Hieracium pilosella	0	0	1	0	[7]

order	genus	species	Synonym/subspecies/alternative spelling	Ascomycota	Basidiomycota	Glomeromycota	Mucoromycotina	References
Asterales	*Saussurea*	*involucrata*		1	0	0	0	95
Asterales	*Solidago*	*canadensis*		0	0	1	0	96
Asterales	*Solidago*	*gigantea*		0	0	1	0	7
Asterales	*Solidago*	*missouriensis*		0	0	1	0	7
Asterales	*Solidago*	*rugosa*		0	0	1	0	7
Asterales	*Solidago*	*virgaurea*		0	0	1	0	7
Asterales	*Sonchus*	*tenerrimus*		0	0	1	0	7
Asterales	*Tanacetum*	*vulgare*		0	0	1	0	7
Asterales	*Taraxacum*	*officinale*		0	0	1	0	7
Asterales	*Tragopogon*	*pratensis*		0	0	1	0	7
Austrobaileyales	*Austrobaileya*	*scandens*		0	0	1	0	7
Boraginales	*Pulmonaria*	*obscura*		0	0	1	0	44
Boraginales	*Trachystemon*	*orientalis*		0	0	1	0	7
Brassicales	*Boscia*	*foetida*		0	0	1	0	7
Brassicales	*Brassica*	*napus*		0	0	1	0	91,97
Brassicales	*Cardamine*	*pratensis*		0	0	1	0	98
Brassicales	*Moringa*	*drouhardii*		0	0	1	0	7
Brassicales	*Moringa*	*hildebrandtii*		0	0	1	0	7
Brassicales	*Moringa*	*oleifera*		0	0	1	0	7
Brassicales	*Noccaea*	*caerulescens*	Thlaspi caerulescens	0	0	1	0	7
Brassicales	*Salvadora*	*persica*		0	0	1	0	99,100
Brassicales	*Subularia*	*aquatica*		0	0	0	0	94
Brassicales	*Thlaspi*	*arvense*		0	0	1	0	7
Caryophyllales	*Arthrocnemum*	*macrostachyum*		0	0	1	0	7
Caryophyllales	*Bistorta*	*vivipara*		1	1	1	0	91,101-105
Caryophyllales	*Dysphania*	*ambrosioides*		0	0	1	0	7
Caryophyllales	*Fagopyrum*	*esculentum*		0	0	1	0	106
Caryophyllales	*Fagopyrum*	*tataricum*		0	0	1	0	7
Caryophyllales	*Guapira*	*discolor*		0	1	0	0	107
Caryophyllales	*Guapira*	*fragrans*		0	1	0	0	107
Caryophyllales	*Phytolacca*	*americana*		0	0	1	0	7
Caryophyllales	*Pisonia*	*aculeata*		0	1	0	0	107
Caryophyllales	*Pisonia*	*albida*		0	1	0	0	107
Caryophyllales	*Pisonia*	*grandis*		0	1	0	0	107
Caryophyllales	*Pisonia*	*sandwicensis*		0	1	0	0	107
Caryophyllales	*Pisonia*	*sechellarum*		0	0	1	0	7
Caryophyllales	*Pisonia*	*taina*		0	1	0	0	107
Caryophyllales	*Polygonum*	*cuspidatum*		0	0	1	0	7
Caryophyllales	*Salicornia*	*europaea*		0	0	1	0	7
Chloranthales	*Hedyosmum*	*goudotianum*		0	0	1	0	108
Commelinales	*Commelina*	*benghalensis*		0	0	1	0	7
Cornales	*Cornus*	*suecica*		0	0	1	0	7
Cucurbitales	*Citrullus*	*colocynthis*		0	0	1	0	7
Cucurbitales	*Gynostemma*	*pentaphyllum*		0	0	1	0	109
Dioscoreales	*Burmannia*	*capitata*		0	0	1	0	7
Dioscoreales	*Dioscorea*	*rotundata*		0	0	1	0	7
Dioscoreales	*Tacca*	*plantaginea*		0	0	1	0	7
Dioscoreales	*Thismia*	*rodwayi*		0	0	1	0	7
Dipsacales	*Knautia*	*arvensis*		0	0	1	0	7
Dipsacales	*Linnaea*	*borealis*		0	0	1	0	7
Ericales	*Aegiceras*	*corniculatum*		0	0	1	0	110
Ericales	*Agarista*	*salicifolia*		0	1	0	0	111
Ericales	*Anagallis*	*arvensis*		0	0	1	0	112
Ericales	*Andromeda*	*polifolia*		1	1	0	0	111,113
Ericales	*Arbutus*	*unedo*		1	1	0	0	111,114,115
Ericales	*Arctostaphylos*	*uva-ursi*		1	1	0	0	116
Ericales	*Calluna*	*vulgaris*		1	1	1	0	10,111,117
Ericales	*Camellia*	*japonica*		0	0	1	0	118
Ericales	*Chimaphila*	*umbellata*		1	1	0	0	119,120
Ericales	*Comarostaphylis*	*arbutoides*		1	1	0	0	121,122
Ericales	*Empetrum*	*nigrum*		1	1	0	0	111,113
Ericales	*Enkianthus*	*campanulatus*		0	0	1	0	7
Ericales	*Enkianthus*	*cernuus*		0	0	1	0	123
Ericales	*Enkianthus*	*nudipes*		0	0	1	0	123
Ericales	*Enkianthus*	*perulatus*		0	0	1	0	123
Ericales	*Enkianthus*	*sikokianus*		0	0	1	0	123
Ericales	*Enkianthus*	*subsessilis*		0	0	1	0	123
Ericales	*Erica*	*carnea*		1	0	0	0	124,125
Ericales	*Erica*	*ciliaris*		0	1	0	0	111
Ericales	*Erica*	*cinerea*		1	1	0	0	10,111
Ericales	*Erica*	*vagans*		0	1	0	0	111
Ericales	*Gaultheria*	*procumbens*		0	1	0	0	111,115
Ericales	*Gaultheria*	*shallon*		1	1	0	0	111,115,126
Ericales	*Orthilia*	*secunda*		1	1	0	0	111,115,119,120
Ericales	*Purdiaea*	*nutans*		0	0	1	0	127
Ericales	*Pyrola*	*minor*		0	1	0	0	120
Ericales	*Pyrola*	*rotundifolia*		1	1	0	0	115,128
Ericales	*Rhododendron*	*decorum*		1	1	0	0	111,129
Ericales	*Rhododendron*	*ferrugineum*		0	1	0	0	111
Ericales	*Rhododendron*	*fortunei*		1	1	0	0	130
Ericales	*Rhododendron*	*groenlandicum*		0	1	0	0	111
Ericales	*Rhododendron*	*racemosum*		0	1	0	0	111
Ericales	*Rhododendron*	*tomentosum*	*Ledum palustre*[131]	0	1	0	0	111
Ericales	*Schizocodon*	*soldanelloides*		1	0	0	0	132
Ericales	*Trientalis*	*europaea*		0	0	1	0	7
Ericales	*Vaccinium*	*myrtillus*		1	1	1	0	10,111,117

order	genus	species	Synonym/subspecies/alternative spelling	Ascomycota	Basidiomycota	Glomeromycota	Mucoromycotina	References
Ericales	*Vaccinium*	*oxycoccos*		1	1	0	0	10,111
Ericales	*Vaccinium*	*poasanum*		0	1	0	0	115
Ericales	*Vaccinium*	*uliginosum*		1	1	0	0	111,113,115
Ericales	*Vaccinium*	*vitis-idaea*		1	1	0	0	111,113
Ericales	*Woollsia*	*pungens*		1	0	0	0	133,134
Fabales	*Acacia*	*mangium*		0	1	1	0	135,136
Fabales	*Afzelia*	*africana*		0	1	0	0	137
Fabales	*Arachis*	*hypogaea*		0	0	1	0	31
Fabales	*Cajanus*	*cajan*		0	0	1	0	7
Fabales	*Caragana*	*korshinskii*		0	0	1	0	7
Fabales	*Delonix*	*regia*		0	0	1	0	138
Fabales	*Dichrostachys*	*cinerea*		0	0	1	0	7
Fabales	*Dicorynia*	*guianensis*		0	0	1	0	7
Fabales	*Eperua*	*falcata*		0	0	1	0	7
Fabales	*Eperua*	*grandiflora*		0	0	1	0	7
Fabales	*Genista*	*cinerea*		0	0	1	0	7
Fabales	*Gliricidia*	*sepium*		0	0	1	0	7
Fabales	*Glycine*	*max*		0	0	1	0	7
Fabales	*Hymenostegia*	*ngouniensis*	*ngounyensis*	0	0	1	0	7
Fabales	*Inga*	*acreana*		0	0	1	0	7
Fabales	*Inga*	*edulis*		0	0	1	0	7
Fabales	*Intsia*	*bijuga*		0	1	0	0	139
Fabales	*Kummerowia*	*striata*		0	0	1	0	140
Fabales	*Lotus*	*brunneri*		0	0	1	0	141
Fabales	*Lotus*	*corniculatus*		0	0	1	0	7
Fabales	*Lotus*	*jacobaeus*		0	0	1	0	7
Fabales	*Lotus*	*japonicus*		0	0	1	0	7
Fabales	*Lupinus*	*latifolius*		0	0	0	0	142
Fabales	*Medicago*	*murex*		0	0	1	0	7
Fabales	*Medicago*	*polymorpha*		0	0	1	0	7
Fabales	*Medicago*	*truncatula*		0	0	1	0	7
Fabales	*Melilotus*	*albus*	*melilotus alba*	0	0	1	0	7
Fabales	*Ononis*	*repens*		0	0	1	0	7
Fabales	*Phaseolus*	*vulgaris*		0	0	1	0	7
Fabales	*Pisum*	*sativum*		0	0	1	0	7
Fabales	*Polygala*	*amara*		0	0	1	0	143
Fabales	*Polygala*	*calcarea*		0	0	1	0	143
Fabales	*Polygala*	*comosa*		0	0	1	0	143
Fabales	*Polygala*	*myrtifolia*		0	0	1	0	143
Fabales	*Polygala*	*rupestris*		0	0	1	0	143
Fabales	*Polygala*	*serpyllifolia*		0	0	1	0	143
Fabales	*Polygala*	*vulgaris*		0	0	1	0	143
Fabales	*Retama*	*sphaerocarpa*		0	0	1	0	7
Fabales	*Robinia*	*pseudoacacia*		0	0	1	0	144
Fabales	*Senegalia*	*senegal*	*Acacia senegal*	0	0	1	0	145
Fabales	*Trifolium*	*pratense*		0	0	1	0	7,146
Fabales	*Trifolium*	*repens*		0	0	1	0	7,146
Fabales	*Trifolium*	*subterraneum*		0	0	1	0	7
Fabales	*Vachellia*	*erioloba*	*Acacia erioloba*	0	0	1	0	7
Fabales	*Vicia*	*faba*		0	0	1	0	7
Fabales	*Vicia*	*hirsuta*		0	0	1	0	7
Fabales	*Vicia*	*sativa*		0	0	1	0	7
Fabales	*Vicia*	*tetrasperma*		0	0	1	0	7
Fabales	*Vigna*	*unguiculata*		0	0	1	0	147
Fagales	*Alnus*	*acuminata*		0	1	0	0	148
Fagales	*Alnus*	*alnobetula*		0	1	0	0	149
Fagales	*Alnus*	*cordata*		0	1	0	0	150
Fagales	*Alnus*	*glutinosa*		1	1	1	0	150-152
Fagales	*Alnus*	*incana*		1	1	1	0	150,151,153
Fagales	*Alnus*	*nitida*		1	1	0	0	154,155
Fagales	*Alnus*	*rubra*		0	1	0	0	156
Fagales	*Betula*	*alleghaniensis*		1	1	0	0	157,158
Fagales	*Betula*	*papyrifera*		1	1	0	0	159
Fagales	*Betula*	*pendula*		1	1	0	0	160,161
Fagales	*Betula*	*platyphylla*		1	1	0	0	162
Fagales	*Betula*	*pubescens*		1	1	0	0	10,163
Fagales	*Carya*	*illinoinensis*		1	1	0	0	164
Fagales	*Castanea*	*dentata*		1	1	0	0	165,166
Fagales	*Castanea*	*mollissima*		1	1	0	0	166,167
Fagales	*Castanea*	*sativa*		0	1	0	0	71
Fagales	*Castanopsis*	*fargesii*		1	1	0	0	168
Fagales	*Casuarina*	*equisetifolia*		1	1	1	0	169,170
Fagales	*Corylus*	*avellana*		1	1	0	0	171
Fagales	*Corylus*	*colurna*		0	1	0	0	64
Fagales	*Fagus*	*grandifolia*		0	1	0	0	172,173
Fagales	*Fagus*	*sylvatica*		1	1	0	0	10,174,175
Fagales	*Juglans*	*regia*		0	1	1	0	176,177
Fagales	*Lophozonia*	*cunninghamii*	*Nothofagus cunninghamii*	1	1	0	0	178
Fagales	*Lophozonia*	*menziesii*	*Nothofagus menziesii*	1	1	0	0	179
Fagales	*Ostrya*	*carpinifolia*		1	1	0	0	171
Fagales	*Quercus*	*agrifolia*		0	1	1	0	180
Fagales	*Quercus*	*alba*		1	1	0	0	181
Fagales	*Quercus*	*cerris*		1	1	0	0	175,182,183
Fagales	*Quercus*	*douglasii*		1	1	1	0	7,181
Fagales	*Quercus*	*fabri*		1	1	0	0	184
Fagales	*Quercus*	*ilex*		1	1	0	0	71,181

order	genus	species	Synonym/subspecies/alternative spelling	Ascomycota	Basidiomycota	Glomeromycota	Mucoromycotina	References
Fagales	*Quercus*	*incana*		1	1	0	0	176,185
Fagales	*Quercus*	*petraea*		1	1	0	0	181
Fagales	*Quercus*	*phellos*		1	1	0	0	186,187
Fagales	*Quercus*	*pubescens*		1	1	0	0	71,188
Fagales	*Quercus*	*robur*		1	1	0	0	175,181,182,189
Fagales	*Quercus*	*rubra*		1	1	1	0	167,181,189-191
Fagales	*Quercus*	*salicina*		0	1	0	0	192
Fagales	*Quercus*	*suber*		1	1	0	0	193
Fagales	*Quercus*	*variabilis*		1	1	0	0	194
Fagales	*Quercus*	*wislizeni*		1	1	0	0	181
Fagales	*Trisyngyne*	*balansae*	*nothofagus*[195]	0	1	0	0	196
Fagales	*Trisyngyne*	*codonandra*	*nothofagus*[195]	0	1	0	0	196
Gentianales	*Cinchona*	*officinalis*		0	0	1	0	7
Gentianales	*Coffea*	*arabica*		0	0	1	0	197
Gentianales	*Faramea*	*occidentalis*		0	0	1	0	7
Gentianales	*Galium*	*album*		0	0	1	0	146
Gentianales	*Galium*	*aparine*		0	0	1	0	7
Gentianales	*Vincetoxicum*	*rossicum*		0	0	1	0	198
Geraniales	*Geranium*	*pratense*		0	0	1	0	7
Lamiales	*Acanthus*	*ilicifolius*		0	0	1	0	199,200
Lamiales	*Ajuga*	*reptans*		0	0	1	0	7
Lamiales	*Avicennia*	*officinalis*		0	0	1	0	200
Lamiales	*Callicarpa*	*americana*		0	0	1	0	201
Lamiales	*Erythranthe*	*guttata*	*Mimulus guttatus*	0	0	1	0	7
Lamiales	*Fraxinus*	*excelsior*		0	0	1	0	7,152
Lamiales	*Glechoma*	*hederacea*		0	0	1	0	7
Lamiales	*Handroanthus*	*ochraceus*	*Tabebuia chrysantha*	0	0	1	0	7
Lamiales	*Lavandula*	*latifolia*		0	0	1	0	7
Lamiales	*Ligustrum*	*vulgare*		0	0	1	0	152
Lamiales	*Littorella*	*uniflora*		0	0	1	0	7,94,202
Lamiales	*Olea*	*europaea*		0	0	1	0	7
Lamiales	*Origanum*	*vulgare*		0	0	1	0	203
Lamiales	*Perilla*	*frutescens*		0	0	1	0	204
Lamiales	*Plantago*	*afra*		0	0	1	0	7
Lamiales	*Plantago*	*asiatica*		0	0	1	0	205
Lamiales	*Plantago*	*lanceolata*		0	0	1	0	7,146,206
Lamiales	*Plantago*	*major*		0	0	1	0	7,146
Lamiales	*Prunella*	*vulgaris*		0	0	1	0	7,38,206
Lamiales	*Rosmarinus*	*officinalis*		0	0	1	0	7
Lamiales	*Tectona*	*grandis*		0	0	1	0	207
Lamiales	*Thymus*	*praecox*		0	0	1	0	123
Lamiales	*Thymus*	*pulegioides*		0	0	1	0	93
Lamiales	*Verbena*	*officinalis*		0	0	1	0	7,208
Lamiales	*Veronica*	*chamaedrys*		0	0	1	0	7,146
Laurales	*Cinnamomum*	*bejolghota*		0	1	0	0	209
Laurales	*Litsea*	*cubeba*		0	0	1	0	7,204
Liliales	*Clintonia*	*borealis*		0	0	1	0	7
Liliales	*Paris*	*incompleta*		0	0	1	0	7
Liliales	*Paris*	*quadrifolia*		0	0	1	0	7
Liliales	*Toxicoscordion*	*venenosum*	*Zigadenus venenosus*	0	0	1	0	38
Liliales	*Trillium*	*grandiflorum*		0	0	1	0	7
Liliales	*Veratrum*	*oxysepalum*		0	0	1	0	7
Malpighiales	*Excoecaria*	*agallocha*		0	0	1	0	199,200,210,211
Malpighiales	*Hieronyma*	*oblonga*	*Hyeronima oblonga*	0	0	1	0	7,212
Malpighiales	*Hypericum*	*maculatum*		0	0	1	0	7
Malpighiales	*Kandelia*	*candel*		0	0	1	0	200,213
Malpighiales	*Mercurialis*	*perennis*		0	0	1	0	214
Malpighiales	*Phyllanthus*	*calycinus*		0	1	1	0	64,215
Malpighiales	*Populus*	*alba*		1	1	1	0	183,216,217
Malpighiales	*Populus*	*balsamifera*		0	1	0	0	218
Malpighiales	*Populus*	*davidiana*		0	1	0	0	219
Malpighiales	*Populus*	*deltoides*		1	1	1	0	186,187,220
Malpighiales	*Populus*	*euphratica*		0	0	1	0	221
Malpighiales	*Populus*	*nigra*		1	1	1	0	152,218,222
Malpighiales	*Populus*	*simonii*		1	1	0	0	223
Malpighiales	*Populus*	*tremula*		1	1	1	0	152,218,224,225
Malpighiales	*Populus*	*trichocarpa*		0	1	1	0	218,226,227
Malpighiales	*Rhizophora*	*apiculata*		0	0	1	0	200
Malpighiales	*Salix*	*alba*		1	1	0	0	228
Malpighiales	*Salix*	*arctica*		1	1	0	0	229
Malpighiales	*Salix*	*herbacea*		1	1	0	0	230
Malpighiales	*Salix*	*polaris*		1	1	0	0	231
Malpighiales	*Salix*	*reinii*		1	1	0	0	232,233
Malpighiales	*Salix*	*reticulata*		1	1	0	0	234
Malpighiales	*Salix*	*tetrasperma*		1	1	0	0	185
Malvales	*Anisoptera*	*costata*		0	1	0	0	235
Malvales	*Dipterocarpus*	*tuberculatus*		0	1	0	0	235
Malvales	*Helianthemum*	*squamatum*		0	0	1	0	7
Malvales	*Hopea*	*odorata*		0	1	0	0	235
Malvales	*Luehea*	*seemannii*		0	0	1	0	7
Malvales	*Shorea*	*guiso*		0	1	0	0	235
Malvales	*Shorea*	*obtusa*		0	1	0	0	235
Malvales	*Tilia*	*americana*		1	0	0	0	188
Malvales	*Tilia*	*cordata*		1	1	1	0	152,236-238
Myrtales	*Alzatea*	*verticillata*		0	0	1	0	7
Myrtales	*Chamerion*	*angustifolium*		0	0	1	0	206

order	genus	species	Synonym/subspecies/alternative spelling	Ascomycota	Basidiomycota	Glomeromycota	Mucoromycotina	References
Myrtales	*Epilobium*	*ciliatum*		0	0	1	0	7
Myrtales	*Eucalyptus*	*camaldulensis*		0	1	0	0	239
Myrtales	*Eucalyptus*	*globulus*		0	1	0	0	239
Myrtales	*Eucalyptus*	*gunnii*		1	1	0	0	240
Myrtales	*Eucalyptus*	*marginata*		0	1	0	0	239,241
Myrtales	*Eucalyptus*	*nitens*		1	1	0	0	240
Myrtales	*Sonneratia*	*alba*		0	0	1	0	200,242
Nymphaeales	*Nymphaea*	*alba*		0	0	0	0	91
Oxalidales	*Oxalis*	*acetosella*		0	0	1	0	7
Oxalidales	*Oxalis*	*stricta*	*Oxalis europaea*	0	0	1	0	243
Petrosaviales	*Japonolirion*	*osense*		0	0	1	0	244
Piperales	*Piper*	*nigrum*		0	0	1	0	245
Poales	*Agrostis*	*capillaris*		0	0	1	0	7
Poales	*Agrostis*	*scabra*		0	0	1	0	7
Poales	*Agrostis*	*stolonifera*		0	0	1	0	7
Poales	*Ammophila*	*arenaria*		0	0	1	0	7
Poales	*Ammophila*	*breviligulata*		0	0	1	0	7
Poales	*Andropogon*	*gerardii*		0	0	1	0	7
Poales	*Anthoxanthum*	*odoratum*		0	0	1	0	146
Poales	*Aristida*	*adscensionis*		0	0	1	0	141
Poales	*Arrhenatherum*	*elatius*		0	0	1	0	7,146
Poales	*Avena*	*barbata*		0	0	1	0	7
Poales	*Bouteloua*	*gracilis*		0	0	1	0	7
Poales	*Brachypodium*	*pinnatum*		0	0	1	0	7
Poales	*Briza*	*media*		0	0	1	0	7
Poales	*Bromus*	*erectus*		0	0	1	0	203
Poales	*Bromus*	*tectorum*		0	0	1	0	7,246
Poales	*Calamagrostis*	*arundinacea*		0	0	1	0	44
Poales	*Cenchrus*	*ciliaris*		0	0	1	0	7
Poales	*Cymbopogon*	*nardus*		0	0	1	0	7
Poales	*Cynosurus*	*cristatus*		0	0	1	0	7
Poales	*Cynosurus*	*echinatus*		0	0	1	0	7
Poales	*Dactylis*	*glomerata*		0	0	1	0	7,146
Poales	*Festuca*	*idahoensis*		0	0	1	0	38
Poales	*Festuca*	*ovina*		0	0	1	0	7,206
Poales	*Festuca*	*pratensis*		0	0	1	0	7
Poales	*Festuca*	*rubra*		0	0	1	0	7
Poales	*Festuca*	*rupicola*		0	0	1	0	7
Poales	*Heteropogon*	*contortus*		0	0	1	0	7
Poales	*Holcus*	*lanatus*		0	0	1	0	7,206
Poales	*Hordeum*	*vulgare*		0	0	1	0	7
Poales	*Juncus*	*bulbosus*		0	0	1	0	7
Poales	*Kobresia*	*myosuroides*		1	1	0	0	247
Poales	*Leymus*	*mollis*	*Elymus mollis*	0	0	1	0	248
Poales	*Lolium*	*multiflorum*		0	0	1	0	146
Poales	*Lolium*	*perenne*		0	0	1	0	7
Poales	*Megathyrsus*	*maximus*	*Panicum maximum*	0	0	1	0	7
Poales	*Melinis*	*repens*	*Rhynchelytrum repens*	0	0	1	0	7
Poales	*Miscanthus*	*sinensis*		0	0	1	0	7
Poales	*Oloptum*	*miliaceum*	*Piptatherum miliaceum*	0	0	1	0	7
Poales	*Oryza*	*sativa*		0	0	1	0	249
Poales	*Panicum*	*virgatum*		0	0	1	0	250,251
Poales	*Paspalum*	*distichum*		0	0	1	0	7
Poales	*Phalaris*	*arundinacea*		0	0	1	0	252
Poales	*Phragmites*	*australis*		0	0	1	0	7
Poales	*Poa*	*pratensis*		0	0	1	0	7
Poales	*Puccinellia*	*distans*		0	0	1	0	7
Poales	*Puccinellia*	*maritima*		0	0	1	0	253
Poales	*Sesleria*	*caerulea*		0	0	1	0	7
Poales	*Setaria*	*pumila*		0	0	1	0	7
Poales	*Sorghum*	*bicolor*		0	0	1	0	7
Poales	*Triraphis*	*mollis*		0	0	1	0	7
Poales	*Trisetum*	*flavescens*		0	0	1	0	146
Poales	*Triticum*	*aestivum*		0	0	1	0	7
Poales	*Typha*	*angustifolia*		0	0	1	0	254
Poales	*Typha*	*latifolia*		0	0	1	0	255,256
Poales	*Zea*	*mays*		0	0	1	0	7,152
Poales	*Zoysia*	*japonica*		0	0	1	0	7
Ranunculales	*Anemone*	*patens*	*Pulsatilla patens*	0	0	1	0	257,258
Ranunculales	*Caltha*	*palustris*		0	0	1	0	7
Ranunculales	*Clematis*	*vitalba*		0	0	1	0	7
Ranunculales	*Ranunculus*	*montanus*		0	0	1	0	93
Ranunculales	*Ranunculus*	*repens*		0	0	1	0	259-261
Ranunculales	*Thalictrum*	*minus*		0	0	1	0	7
Rosales	*Cecropia*	*obtusa*		0	0	1	0	7
Rosales	*Cercocarpus*	*ledifolius*		1	1	0	0	262
Rosales	*Crataegus*	*monogyna*		0	0	1	0	152
Rosales	*Dryas*	*integrifolia*		1	1	0	0	229
Rosales	*Dryas*	*octopetala*		1	1	0	0	234
Rosales	*Fragaria*	*vesca*		0	0	1	0	7
Rosales	*Geum*	*rivale*		0	0	1	0	7
Rosales	*Malus*	*hupehensis*		0	0	1	0	263
Rosales	*Malus*	*micromalus*		0	0	1	0	264
Rosales	*Malus*	*sieboldii*		0	0	1	0	265
Rosales	*Pilea*	*pumila*		0	0	1	0	7
Rosales	*Potentilla*	*erecta*		0	0	1	0	146

order	genus	species	Synonym/subspecies/alternative spelling	Ascomycota	Basidiomycota	Glomeromycota	Mucoromycotina	References
Rosales	*Prunus*	*africana*		0	0	1	0	7
Rosales	*Prunus*	*persica*		0	0	1	0	7
Rosales	*Pyrus*	*pyrifolia*		0	0	1	0	205
Rosales	*Rosa*	*multiflora*		0	0	1	0	27
Rosales	*Rubus*	*parvifolius*		0	0	1	0	27
Rosales	*Rubus*	*saxatilis*		0	0	1	0	7
Rosales	*Sorbus*	*aucuparia*		0	0	1	0	152
Rosales	*Ulmus*	*americana*		1	1	0	0	266
Sapindales	*Acer*	*macrophyllum*		0	0	1	0	267
Sapindales	*Acer*	*platanoides*		0	0	1	0	152,267
Sapindales	*Acer*	*pseudoplatanus*		0	0	1	0	7,267
Sapindales	*Cedrela*	*odorata*		0	0	1	0	212
Sapindales	*Citrus*	*maxima*		0	1	1	0	268,269
Sapindales	*Cneorum*	*tricoccon*		0	0	1	0	7
Sapindales	*Dimocarpus*	*longan*		0	1	1	0	270,271
Sapindales	*Dodonaea*	*viscosa*		0	0	1	0	7
Sapindales	*Guarea*	*pterorhachis*		0	0	1	0	7
Sapindales	*Mangifera*	*indica*		0	1	1	0	270,272,273
Sapindales	*Orixa*	*japonica*		0	0	1	0	7
Sapindales	*Phellodendron*	*amurense*		0	0	1	0	7
Sapindales	*Ruta*	*chalepensis*		0	0	1	0	7
Sapindales	*Santiria*	*trimera*		0	0	1	0	7
Sapindales	*Swietenia*	*macrophylla*		0	0	1	0	7
Sapindales	*Tetragastris*	*panamensis*		0	0	1	0	7
Saxifragales	*Saxifraga*	*oppositifolia*		0	0	1	0	7
Solanales	*Ipomoea*	*pes-caprae*		0	0	1	0	7
Solanales	*Lycium*	*barbarum*		0	0	1	0	274,275
Solanales	*Montinia*	*caryophyllacea*		0	0	1	0	89
Solanales	*Solanum*	*tuberosum*		0	0	1	0	276
Vitales	*Vitis*	*vinifera*		0	0	1	0	7
Zingiberales	*Musa*	*acuminata*		0	0	1	0	277
Zingiberales	*Zingiber*	*officinale*		0	0	1	0	91,278
**Tracheophyta, Gymnosperm**								
Araucariales	*Afrocarpus*	*falcatus*	*Podocarpus falcatus*[279]	0	0	1	0	280
Araucariales	*Araucaria*	*angustifolia*		0	0	1	0	281,282
Araucariales	*Dacrycarpus*	*dacrydioides*		0	0	1	0	7
Araucariales	*Podocarpus*	*oleifolius*		0	0	1	0	7
Araucariales	*Prumnopitys*	*ferruginea*		0	0	1	0	7
Araucariales	*Prumnopitys*	*montana*		0	0	1	0	283,284
Araucariales	*Prumnopitys*	*taxifolia*		0	0	1	0	7
Cupressales	*Austrocedrus*	*chilensis*		0	0	1	0	285
Cupressales	*Chamaecyparis*	*obtusa*		0	0	1	0	7
Cupressales	*Sequoiadendron*	*giganteum*		0	0	1	0	7
Cupressales	*Taxus*	*baccata*		0	1	1	0	7,286
Ephedrales	*Ephedra*	*fragilis*		0	0	1	0	7
Ginkgoales	*Ginkgo*	*biloba*		0	0	1	0	287
Gnetales	*Gnetum*	*gnemon*		0	1	0	0	225
Pinales	*Abies*	*alba*		1	1	0	0	288
Pinales	*Abies*	*balsamea*	*Abies balsamifera*	1	1	0	0	289
Pinales	*Abies*	*homolepis*		1	1	0	0	290
Pinales	*Abies*	*magnifica*		0	1	0	0	291
Pinales	*Abies*	*nordmanniana*		0	1	0	0	286
Pinales	*Abies*	*religiosa*		1	1	0	0	292,293
Pinales	*Cedrus*	*atlantica*		0	1	0	0	286
Pinales	*Cedrus*	*deodara*		1	1	0	0	294,295
Pinales	*Keteleeria*	*davidiana*		1	1	0	0	296
Pinales	*Larix*	*decidua*		1	1	0	0	297
Pinales	*Larix*	*gmelinii*		1	1	0	0	298
Pinales	*Larix*	*kaempferi*		1	1	0	0	290
Pinales	*Picea*	*abies*		1	1	0	0	299
Pinales	*Picea*	*crassifolia*		1	1	0	0	300
Pinales	*Picea*	*glauca*		1	1	0	0	301
Pinales	*Picea*	*jezoensis*		0	1	0	0	290
Pinales	*Picea*	*mariana*		1	1	0	0	302
Pinales	*Picea*	*rubens*		1	0	0	0	303
Pinales	*Picea*	*sitchensis*		0	1	0	0	304
Pinales	*Picea*	*smithiana*		0	1	0	0	305
Pinales	*Pinus*	*albicaulis*		1	1	0	0	306
Pinales	*Pinus*	*banksiana*		1	1	0	0	307
Pinales	*Pinus*	*canariensis*		0	1	0	0	308
Pinales	*Pinus*	*caribaea*		1	1	0	0	309,310
Pinales	*Pinus*	*cembra*		1	1	0	0	311
Pinales	*Pinus*	*contorta*		1	1	0	1	312-314
Pinales	*Pinus*	*densiflora*		1	1	0	0	315-317
Pinales	*Pinus*	*edulis*		1	1	0	0	318,319
Pinales	*Pinus*	*elliottii*		1	1	0	0	320
Pinales	*Pinus*	*halepensis*		0	1	0	0	71,308,321
Pinales	*Pinus*	*jeffreyi*		0	1	0	0	322
Pinales	*Pinus*	*kesiya*		1	1	0	0	323,324
Pinales	*Pinus*	*koraiensis*		1	1	0	0	325
Pinales	*Pinus*	*lambertiana*		1	1	0	0	326
Pinales	*Pinus*	*longaeva*		1	1	0	0	327
Pinales	*Pinus*	*massoniana*		1	1	0	0	184,328
Pinales	*Pinus*	*montezumae*		1	1	0	0	329
Pinales	*Pinus*	*mugo*		1	1	0	0	330
Pinales	*Pinus*	*muricata*		1	1	0	0	331,332

order	genus	species	Synonym/subspecies/alternative spelling	Ascomycota	Basidiomycota	Glomeromycota	Mucoromycotina	References
Pinales	*Pinus*	*nigra*		1	1	0	0	189
Pinales	*Pinus*	*parviflora*		0	1	0	0	290
Pinales	*Pinus*	*patula*		1	1	0	0	333,334
Pinales	*Pinus*	*pinaster*		1	1	0	0	71,308,335
Pinales	*Pinus*	*pinea*		1	1	0	0	308,336
Pinales	*Pinus*	*ponderosa*		1	1	0	0	313,337
Pinales	*Pinus*	*pumila*		0	1	0	0	338
Pinales	*Pinus*	*radiata*		1	1	0	0	308,320
Pinales	*Pinus*	*roxburghii*		0	1	0	0	339
Pinales	*Pinus*	*sabiniana*		1	1	0	0	340
Pinales	*Pinus*	*strobus*		1	1	0	0	341
Pinales	*Pinus*	*sylvestris*		1	1	0	0	71,189,308
Pinales	*Pinus*	*tabuliformis*		1	1	0	0	223
Pinales	*Pinus*	*taeda*		1	1	0	0	342,343
Pinales	*Pinus*	*thunbergii*		1	1	0	0	344
Pinales	*Pinus*	*virginiana*		1	1	0	0	345,346
Pinales	*Pinus*	*wallichiana*		1	1	0	0	295,347,348
Pinales	*Pseudotsuga*	*menziesii*		1	1	0	0	159,326,332,345
Pinales	*Tsuga*	*canadensis*		1	1	0	0	121,158,349
Pinales	*Tsuga*	*mertensiana*		1	1	0	0	345,350
**Tracheophyta, Lycopodiopsida**								
Isoetales	*Isoetes*	*echinospora*		0	0	1	1	94
Isoetales	*Isoetes*	*lacustris*		0	0	1	0	94
Lycopodiales	*Dendrolycopodium*	*dendroideum*		0	0	0	0	351
Lycopodiales	*Diphasiastrum*	*alpinum*		1	1	1	0	117
Lycopodiales	*Huperzia*	*appressa*		0	0	0	0	351
Lycopodiales	*Huperzia*	*lucidula*		0	0	0	0	351
Lycopodiales	*Lycopodiella*	*inundata*		0	0	0	1	351
Lycopodiales	*Lycopodiella*	*lateralis*		0	0	0	1	351
Lycopodiales	*Lycopodium*	*annotinum*		0	0	0	1	351
Lycopodiales	*Lycopodium*	*clavatum*		0	0	1	1	7,352
Lycopodiales	*Lycopodium*	*fastigiatum*		0	0	0	1	351
Lycopodiales	*Lycopodium*	*volubile*		0	0	0	0	351
Lycopodiales	*Palhinhaea*	*cernua*	*Lycopodium cernuum*[353]	0	0	1	0	351
Lycopodiales	*Phlegmariurus*	*phlegmaria*		0	0	1	0	351
Selaginellales	*Selaginella*	*kraussiana*		0	0	1	0	351
Selaginellales	*Selaginella*	*selaginoides*		0	0	1	0	351
**Tracheophyta, Polypodiopsida**								
Cyatheales	*Dicksonia*	*squarrosa*		0	0	1	0	354,355
Equisetales	*Equisetum*	*fluviatile*		0	0	0	0	91
Gleicheniales	*Diplopterygium*	*glaucum*		0	0	1	0	356
Gleicheniales	*Gleichenia*	*microphylla*		0	0	1	0	351
Marattiales	*Ptisana*	*purpurascens*		0	0	1	0	351
Ophioglossales	*Botrychium*	*virginianum*		0	0	1	0	7
Ophioglossales	*Ophioglossum*	*costatum*		0	0	1	0	351
Ophioglossales	*Ophioglossum*	*vulgatum*		0	0	1	0	351
Osmundales	*Osmunda*	*regalis*		0	0	1	0	7
Polypodiales	*Anogramma*	*leptophylla*		0	0	1	1	2,351
Polypodiales	*Athyrium*	*yokoscense*		0	0	1	0	7
Polypodiales	*Nephrolepis*	*hirsutula*		0	0	1	0	351
Polypodiales	*Polystichum*	*acrostichoides*		0	0	1	0	7
Polypodiales	*Triplophyllum*	*vogelii*		0	0	1	0	7
Psilotales	*Marsilea*	*crenata*		0	0	0	0	357
Psilotales	*Psilotum*	*nudum*		0	0	1	0	7,351
Psilotales	*Tmesipteris*	*elongata*		0	0	1	0	351
Psilotales	*Tmesipteris*	*tannensis*		0	0	1	0	2,7

1 Desirò, A., Duckett, J. G., Pressel, S., Villarreal, J. C. & Bidartondo, M. I. Fungal symbioses in hornworts: a chequered history. *Proceedings of the Royal Society of London B: Biological Sciences* **280**, 20130207-20130207 (2013).

2 Bidartondo, M. I. *et al.* The dawn of symbiosis between plants and fungi. *Biol Letters* **7**, 574-577 (2011).

3 Duff, R. J., Villarreal, J. C., Caagill, D. C. & Renzaglia, K. S. Progress and challenges toward developing a phylogeny and classification of the hornworts. *The Bryologist* **110**, 214-243 (2007).

4 Crandall-Stotler, B. J., Stotler, R. E., Doyle, W. T. & Forrest, L. L. Chapter Nineteen: Phaeoceros proskaueri sp. nov., a New Species of the Phaeoceros hallii (Austin) Prosk.-Phaeoceros pearsonii (M. Howe) Prosk. Complex and the Systematic Affinities of Paraphymatoceros Hässel.". *Fieldiana Botany* **47**, 213-238 (2008).

5 Pressel, S., Bidartondo, M. I., Ligrone, R. & Duckett, J. G. Fungal symbioses in bryophytes : New insights in the Twenty First Century. (2010).

6 Ligrone, R. *et al.* Glomeromycotean associations in liverworts: A molecular, cellular, and taxonomic analysis. *American Journal of Botany* **94**, 1756-1777 (2007).

7 Opik, M. *et al.* The online database MaarjAM reveals global and ecosystemic distribution patterns in arbuscular mycorrhizal fungi (Glomeromycota). *New Phytol* **188**, 223-241 (2010).

8 Bidartondo, M. I. & Duckett, J. G. Conservative ecological and evolutionary patterns in liverwort-fungal symbioses. *Proceedings. Biological sciences / The Royal Society* **277**, 485-492 (2010).

9 Pressel, S., Ligrone, R. & Duckett, J. G. Chapter Six: The Ascomycete Rhizoscyphus ericae Elicits a Range of Host Responses in the Rhizoids of Leafy Liverworts: An Experimental and Cytological Analysis. *Fieldiana Botany* **47**, 59-59 (2008).

10 Duckett, J. G. & Read, D. J. the Use of the Fluorescent Dye, 3,3'-Dihexyloxacarbocyanine Iodide, for Selective Staining of Ascomycete Fungi Associated With Liverwort Rhizoids and Ericoid Mycorrhizal Roots. *New Phytol* **118**, 259-272 (1991).

11 Kottke, I. *et al.* Heterobasidiomycetes form symbiotic associations with hepatics: Jungermanniales have sebacinoid mycobionts while Aneura pinguis (Metzgeriales) is associated with a Tulasnella species. *Mycological Research* **107**, 957-968 (2003).

12 Upson, R., Read, D. & Newsham, K. Widespread association between the ericoid mycorrhizal fungus Rhizoscyphus ericae and a leafy liverwort in the maritime and sub-Antarctic. *New Phytol* **176**, 460-471 (2007).

13 Steere, W. C. Notes on Michigan bryophytes - IV. *The Bryologist* **45**, 153-172 (1942).

14 Duckett, J. G., Russell, J. & Ligrone, R. Basidiomycetous endophytes in jungermannialean (leafy) liverworts have novel cytology and species-specific host ranges: a cytological and experimental study. *Canadian Journal of Botany* **84**, 1075-1093 (2006).

15 Bakalin, V. A., Tigishvili, K. & Arutinov, G. A new checklist of the liverworts and hornworts of Georgia (Caucasus). *Botanica Pacifica* **5**, 69-78 (2016).

16 Söderström, L., Roo, R. d. & Hedderson, T. Taxonomic novelties resulting from recent reclassification of the Lophoziaceae/ Scapaniaceae clade. *Phytotaxa* **3**, 47-53 (2010).

17 Pressel, S., Ligrone, R., Duckett, J. G. & Davis, E. C. A novel ascomycetous endophytic association in the rhizoids of the leafy liverwort family, Schistochilaceae (Jungermanniidae, Hepaticopsida). *American Journal of Botany* **95**, 531-541 (2008).

18 Kürschner, H. Amendments to the bryophyte flora of Lebanon, based on collections of C. B. Arzeni 1962–1964. *The Bryologist* **113**, 717-720 (2010).

19 Bakalin, V. Notes on Lophozia VI. Taxonomy and distribution of Lophozia and Schistochilopsis (Lophoziaceae) in North America north of Mexico. *The Bryologist* **114**, 298-315 (2011).

20 Evans, A. W. Notes on North American Hepaticae. IV. *The Bryologist* **16**, 49-55 (1913).

21 Wickett, N. J. & Goffinet, B. Origin and relationships of the myco-heterotrophic liverwort Cryptothallus mirabilis Malmb. (Metzgeriales, Marchantiophyta). *Botanical Journal of the Linnean Society* **156**, 1-12 (2008).

22 Krause, C., Garnica, S., Bauer, R. & Nebel, M. Aneuraceae (Metzgeriales) and tulasnelloid fungi (Basidiomycota) - a model for early steps in fungal symbiosis. *Fungal Biol-Uk* **115**, 839-851 (2011).

23 Preußing, M. *et al.* New insights in the evolution of the liverwort family aneuraceae (Metzgeriales, Marchantiophyta), with emphasis on the genus Lobatiriccardia. *Taxon* **59**, 1424-1440 (2010).

24 Fuselier, L. C. *et al.* The status and phylogeography of the liverwort genus Apometzgeria Kuwah. (Metzgeriaceae). *The Bryologist* **114**, 92-101 (2011).

25 Brundrett, M. C. Mycorrhizal associations and other means of nutrition of vascular plants: Understanding the global diversity of host plants by resolving conflicting information and developing reliable means of diagnosis. *Plant Soil* **320**, 37-77 (2009).

26 Campagnac, E. *et al.* Differential effects of fenpropimorph and fenhexamid, two sterol biosynthesis inhibitor fungicides, on arbuscular mycorrhizal development and sterol metabolism in carrot roots. *Phytochemistry* **69**, 2912-2919 (2008).

27 Ahulu, E. M., Andoh, H. & Nonaka, M. Host-related variability in arbuscular mycorrhizal fungal structures in roots of Hedera rhombea, Rubus parvifolius, and Rosa multiflora under controlled conditions. *Mycorrhiza* **17**, 93-101 (2007).

28 Kil, Y. J., Eo, J. K., Lee, E. H. & Eom, A. H. Root age-dependent changes in arbuscular mycorrhizal fungal communities colonizing roots of Panax ginseng. *Mycobiology* **42**, 416-421 (2014).

29 Suárez, J. P. *et al.* Diverse tulasnelloid fungi form mycorrhizas with epiphytic orchids in an Andean cloud forest. *Mycological Research* **110**, 1257-1270 (2006).

30 Perner, H., Schwarz, D. & George, E. Effect of mycorrhizal inoculation and compost supply on growth and nutrient uptake of young leek plants grown on peat-based substrates. *HortScience* **41**, 628-632 (2006).

31 Lee, J., Lee, S. & Young, J. P. W. Improved PCR primers for the detection and identification of arbuscular mycorrhizal fungi. *FEMS Microbiology Ecology* **65**, 339-349 (2008).

32 Girlanda, M. *et al.* Photosynthetic Mediterranean meadow orchids feature partial mycoheterotrophy and specific mycorrhizal associations1. *American Journal of Botany* **98**, 1148-1163 (2011).

33 Ercole, E. *et al.* Temporal variation in mycorrhizal diversity and carbon and nitrogen stable isotope abundance in the wintergreen meadow orchid Anacamptis morio. *New Phytol* **205**, 1308-1319 (2015).

34 Roy, M. *et al.* Two mycoheterotrophic orchids from Thailand tropical dipterocarpacean forests associate with a broad diversity of ectomycorrhizal fungi. *BMC biology* **7**, 51-51 (2009).

35 Ma, M., Tan, T. K. & Wong, S. M. Identification and molecular phylogeny of Epulorhiza isolates from tropical orchids. *Mycological research* **107**, 1041-1049 (2003).

36 Yergeau, E., Vujanovic, V. & St-Arnaud, M. Changes in communities of Fusarium and arbuscular mycorrhizal fungi as related to different asparagus cultural factors. *Microbial Ecology* **52**, 104-113 (2006).

37 Palenzuela, J., Barea, J.-M., Ferrol, N. & Oehl, F. Ambispora granatensis, a new arbuscular mycorrhizal fungus, associated with Asparagus officinalis in Andalucia (Spain). *Mycologia* **103**, 333-340 (2010).

38 Phillips, W. S. *Drivers of arbuscular mycorrhizal fungal community composition in roots: hosts, neighbors, and environment*, (2012).

39 Sakamoto, Y., Yokoyama, J. & Maki, M. Mycorrhizal diversity of the orchid Cephalanthera longibracteata in Japan. *Mycoscience* **56**, 183-189 (2015).

40 Julou, T. *et al.* Mixotrophy in orchids: Insights from a comparative study of green individuals and nonphotosynthetic individuals of Cephalanthera damasonium. *New Phytol* **166**, 639-653 (2005).

41 Bidartondo, M. I. & Read, D. J. Fungal specificity bottlenecks during orchid germination and development. *Mol Ecol* **17**, 3707-3716 (2008).

42 Bidartondo, M. I., Burghardt, B., Gebauer, G., Bruns, T. D. & Read, D. J. Changing partners in the dark: isotopic and molecular evidence of ectomycorrhizal liaisons between forest orchids and trees. *Proceedings. Biological sciences / The Royal Society* **271**, 1799-1806 (2004).

43 Abadie, J.-C. *et al.* Cephalanthera longifolia (Neottieae, Orchidaceae) is mixotrophic: a comparative study between green and nonphotosynthetic individuals. *Canadian Journal of Botany* **84**, 1462-1477 (2006).

44 Saks, Ü. *et al.* Root-colonizing and soil-borne communities of arbuscular mycorrhizal fungi in a temperate forest understorey 1. *NRC Research Press* **285**, 277-285 (2014).

45 Zimmer, K., Meyer, C. & Gebauer, G. The ectomycorrhizal specialist orchid Corallorhiza trifida is a partial myco-heterotroph. *New Phytol* **178**, 395-400 (2008).

46 Waterman, R. J. *et al.* The effects of above- and belowground mutualisms on orchid speciation and coexistence. *The American naturalist* **177**, E54-E68 (2011).

47 Yagame, T., Funabiki, E., Nagasawa, E., Fukiharu, T. & Iwase, K. Identification and symbiotic ability of Psathyrellaceae fungi isolated from a photosynthetic orchid, Cremastra appendiculata (Orchidaceae). *American Journal of Botany* **100**, 1823-1830 (2013).

48 Jiang, J. H., Lee, Y. I., Cubeta, M. A. & Chen, L. C. Characterization and colonization of endomycorrhizal Rhizoctonia fungi in the medicinal herb Anoectochilus formosanus (Orchidaceae). *Mycorrhiza* **25**, 431-445 (2015).

49 Jiang, J. H., Fang, C. C., Tam, S. L., Shiu, J. F. & Chen, L. C. *Mycorrhizal specificity of Rhizoctonia complex from Orchidaceae in Taiwan (JX514373 - JX514396, KJ573103)*, <http://www.ncbi.nlm.nih.gov/> (2012).

50 Yang, K. *et al. Isolation and identification of Cymbidium plants mycorrhizal fungi (Genbank accession: EF393621)*, <www.ncbi.nlm.nih.gov> (2007).

51 Wu, J. *et al.* Rhizoctonia fungi enhance the growth of the endangered orchid Cymbidium goeringii. *Botany* **88**, 20-29 (2010).

52 Ogura-Tsujita, Y., Yokoyama, J., Miyoshi, K. & Yukawa, T. Shifts in mycorrhizal fungi during the evolution of autotrophy to mycoheterotrophy in Cymbidium (Orchidaceae). *American Journal of Botany* **99**, 1158-1176 (2012).

53 Shefferson, R. P. *et al.* The evolutionary history of mycorrhizal specificity among lady's slipper orchids. *Evolution* **61**, 1380-1390 (2007).

54 Shefferson, R. P., Weiß, M., Kull, T. & Taylor, D. L. High specificity generally characterizes mycorrhizal association in rare lady's slipper orchids, genus Cypripedium. *Mol Ecol* **14**, 613-626 (2005).

55 Yuan, L., Yang, Z. L., Li, S. Y., Hu, H. & Huang, J. L. Mycorrhizal specificity, preference, and plasticity of six slipper orchids from South Western China. *Mycorrhiza* **20**, 559-568 (2010).

56 Xiaohua, J., Singchi, C. & Yibo, L. Taxonomic revision of Dendrobium moniliforme complex (Orchidaceae). *Scientia Horticulturae* **120**, 143-145 (2009).

57 Tan, X. M. *et al.* In vitro seed germination and seedling growth of an endangered epiphytic orchid, Dendrobium officinale, endemic to China using mycorrhizal fungi (Tulasnella sp.). *Scientia Horticulturae* **165**, 62-68 (2014).

58 Xing, X. *et al.* Specificity and preference of mycorrhizal associations in two species of the genus Dendrobium (Orchidaceae). *Mycorrhiza* **23**, 317-324 (2013).

59 Chen, J., Wang, H. & Guo, S. X. Isolation and identification of endophytic and mycorrhizal fungi from seeds and roots of Dendrobium (Orchidaceae). *Mycorrhiza* **22**, 297-307 (2012).

60 Bonnardeaux, Y. *et al.* Diversity of mycorrhizal fungi of terrestrial orchids: compatibility webs, brief encounters, lasting relationships and alien invasions. *Mycological Research* **111**, 51-61 (2007).

61 Sommer, J. *et al.* Limited carbon and mineral nutrient gain from mycorrhizal fungi by adult Australian Orchids. *American Journal of Botany* **99**, 1133-1145 (2012).

62 Riofrío, M. L. *et al.* Mycorrhizal preferences and fine spatial structure of the epiphytic orchid Epidendrum rhopalostele. *American Journal of Botany* **100**, 2339-2348 (2013).

63 Teššitelová, T., Teššitel, J., Jersáková, J., Říhová, G. & Selosse, M. A. Symbiotic germination capability of four epipactis species (orchidaceae) is broader than expected from adult ecology. *American Journal of Botany* **99**, 1020-1032 (2012).

64 Weiss, M., Selosse, M. A., Rexer, K. H., Urban, A. & Oberwinkler, F. Sebacinales: a hitherto overlooked cosm of heterobasidiomycetes with a broad mycorrhizal potential. *Mycol Res* **108**, 1003-1010 (2004).

65 Illyes, Z. *et al.* Changes in the diversity of the mycorrhizal fungi of orchids as a function of the water supply of the habitat. *Journal of Applied Botany and Food Quality* **83**, 28-36 (2009).

66 Shefferson, R. P. *et al.* Evolution of host breadth in broad interactions: Mycorrhizal specificity in East Asian and North American rattlesnake plantains (Goodyera spp.) and their fungal hosts. *Mol Ecol* **19**, 3008-3017 (2010).

67 McCormick, M. K., Whigham, D. F. & O'Neill, J. Mycorrhizal diversity in photosynthetic terrestrial orchids. *New Phytol* **163**, 425-438 (2004).

68 Těšitelová, T. *et al.* Ploidy-specific symbiotic interactions: Divergence of mycorrhizal fungi between cytotypes of the Gymnadenia conopsea group (Orchidaceae). *New Phytol* **199**, 1022-1033 (2013).

69 Stark, C., Babik, W. & Durka, W. Fungi from the roots of the common terrestrial orchid Gymnadenia conopsea. *Mycological Research* **113**, 952-959 (2009).

70 Otero, J. T., Ackerman, J. D. & Bayman, P. Diversity and host specificity of endophytic Rhizoctonia-like fungi from tropical orchids. *American Journal of Botany* **89**, 1852-1858 (2002).

71 Girlanda, M. *et al.* Inefficient photosynthesis in the Mediterranean orchid Limodorum abortivum is mirrored by specific association to ectomycorrhizal Russulaceae. *Mol Ecol* **15**, 491-504 (2006).

72 Shimura, H. *et al.* Characterization of mycorrhizal fungi isolated from the threatened Cypripedium macranthos in a northern island of Japan: Two phylogenetically distinct fungi associated with the orchid. *Mycorrhiza* **19**, 525-534 (2009).

73 Wiegand, K. M. A Revision of the Genus Listera. *Bulletin of the Torrey Botanical Club* (1899).

74 Těšitelová, T. *et al.* Two widespread green Neottia species (Orchidaceae) show mycorrhizal preference for Sebacinales in various habitats and ontogenetic stages. *Mol Ecol* **24**, 1122-1134 (2015).

75 Selosse, M. A., Weiß, M., Jany, J. L. & Tillier, A. Communities and populations of sebacinoid basidiomycetes associated with the achlorophyllous orchid Neottia nidus-avis (L.) L.C.M. Rich. and neighbouring tree ectomycorrhizae. *Mol Ecol* **11**, 1831-1844 (2002).

76 Nomura, N. *et al.* The rare terrestrial orchid Nervilia nipponica consistently associates with a single group of novel mycobionts. *Journal of Plant Research* **126**, 613-623 (2013).

77 Gebauer, G., Preiss, K. & Gebauer, A. C. Partial mycoheterotrophy is more widespread among orchids than previously assumed. *New Phytol* **211**, 11-15 (2016).

78 Schatz, B. *et al.* A case study of modified interactions with symbionts in a hybrid mediterranean orchid. *American Journal of Botany* **97**, 1278-1288 (2010).

79 Jacquemyn, H., Honnay, O., Cammue, B. P. A., Brys, R. & Lievens, B. Low specificity and nested subset structure characterize mycorrhizal associations in five closely related species of the genus Orchis. *Mol Ecol* **19**, 4086-4095 (2010).

80 Shan, X. C., Liew, E. C. Y., Weatherhead, M. a. & Hodgkiss, I. J. Characterization and taxonomic placement of Rhizoctonia-like endophytes from orchid roots. *Mycologia* **94**, 230-239 (2002).

81 Shu Fen, C., Chih Hsing, Y., Chen Han, J. & Doris, C. N. C. Growth and development of Phaius tankervilleae (Banks) Blume when inoculated with orchid mycorrhizal fungi. *African Journal of Agricultural Research* **7**, 5644-5652 (2012).

82 Bateman, R. M. *et al.* Speciation via floral heterochrony and presumed mycorrhizal host switching of endemic butterfly orchids on the Azorean archipelago. *American Journal of Botany* **101**, 979-1001 (2014).

83 Kohout, P., Těšitelová, T., Roy, M., Vohník, M. & Jersáková, J. A diverse fungal community associated with Pseudorchis albida (Orchidaceae) roots. *Fungal Ecology* **6**, 50-64 (2013).

84 Irwin, M. J., Bougoure, J. J. & Dearnaley, J. D. W. Pterostylis nutans (Orchidaceae) has a specific association with two Ceratobasidium root-associated fungi across its range in eastern Australia. *Mycoscience* **48**, 231-239 (2007).

85 Ormerod, P. Orchidaceous additions to the Philippine flora (II). *Taiwania* **53**, 157-164 (2008).

86 Ercole, E. *et al.* Cryopreservation of orchid mycorrhizal fungi: a tool for the conservation of endangered species. *Journal of microbiological methods* **93**, 134-137 (2013).

87 Mosquera-Espinosa, A. T., Bayman, P. & Otero, J. T. Ceratobasidium como hongo micorrízico de orquídeas en Colombia. *Acta Agronómica* **59**, 316-326 (2010).

88 Martos, F. *et al.* Independent recruitment of saprotrophic fungi as mycorrhizal partners by tropical achlorophyllous orchids. *New Phytol* **184**, 668-681 (2009).

89 Gazol, A. *et al.* Impact of alien pines on local arbuscular mycorrhizal fungal communities—evidence from two continents. *FEMS Microbiology Ecology* **92**, fiw073-fiw073 (2016).

90 Torrecillas, E. *et al.* Modularity reveals the tendency of arbuscular mycorrhizal fungi to interact differently with generalist and specialist plant species in gypsum soils. *Appl Environ Microb* **80**, 5457-5466 (2014).

91 Wang, B. & Qiu, Y. L. Phylogenetic distribution and evolution of mycorrhizas in land plants. *Mycorrhiza* **16**, 299-363 (2006).

92 Del Fabbro, C. & Prati, D. Early responses of wild plant seedlings to arbuscular mycorrhizal fungi and pathogens. *Basic and Applied Ecology* **15**, 534-542 (2014).

93 Sýkorová, Z., Wiemken, A. & Redecker, D. Cooccurring Gentiana verna and Gentiana acaulis and their neighboring plants in two Swiss upper montane meadows harbor distinct arbuscular mycorrhizal fungal communities. *Appl Environ Microb* **73**, 5426-5434 (2007).

94 Kohout, P. *et al.* Surprising spectra of root-associated fungi in submerged aquatic plants. *FEMS Microbiol Ecol* **80**, 216-235 (2012).

95 Wu, L. & Guo, S. Interaction between an isolate of dark-septate fungi and its host plant Saussurea involucrata. *Mycorrhiza* **18**, 79-85 (2008).

96 Yuan, Y. *et al.* An invasive plant promotes its arbuscular mycorrhizal symbioses and competitiveness through its secondary metabolites: Indirect evidence from activated carbon. *Plos One* **9** (2014).

97 Higo, M., Isobe, K. & Ishii, R. *Community structure of indigenous arbuscular mycorrhizal fungi in the colonized roots of various winter crops (Genbank accession: AB548524.1 through AB548569.1)*, <www.ncbi.nlm.nih.gov> (2011).

98 Renker, C., Heinrichs, J., Kaldorf, M. & Buscot, F. o. Combining nested PCR and restriction digest of the internal transcribed spacer region to characterize arbuscular mycorrhizal fungi on roots from the field. *Mycorrhiza* **13**, 191-198 (2003).

99 Symanczik, S., Blaszkowski, J., Chwat, G., Boller, T., W., A. & Al-Yahya'Ei, M. N. Three new species of arbuscular mycorrhizal fungi discovered at one location in a desert of Oman: Diversispora omaniana, Septoglomus nakheelum and Rhizophagus arabicus. *Mycologia* (2014).

100 Al-Yahya'ei, M. N. *et al.* Unique arbuscular mycorrhizal fungal communities uncovered in date palm plantations and surrounding desert habitats of Southern Arabia. *Mycorrhiza* **21**, 195-209 (2011).

101 Brevik, A., Moreno-Garcia, J. & Wenelczyk, J. Diversity of fungi associated with Bistorta vivipara (L.) Delarbre root systems along a local chronosequence on Svalbard. *Agarica* **29**, 15-26 (2010).

102 Garnica, S., Riess, K., Bauer, R., Oberwinkler, F. & Weiß, M. Phylogenetic diversity and structure of sebacinoid fungi associated with plant communities along an altitudinal gradient. *FEMS Microbiology Ecology* **83**, 265-278 (2013).

103 BLAALID, R. *et al.* Changes in the root-associated fungal communities along a primary succession gradient analysed by 454 pyrosequencing. *Mol Ecol* (2012).

104 Kauserud, H., Kumar, S., Brysting, A. K., Nordén, J. & Carlsen, T. High consistency between replicate 454 pyrosequencing analyses of ectomycorrhizal plant root samples. *Mycorrhiza* **22**, 309-315 (2012).

105 Eriksen, M., Bjureke, K. E. & Dhillion, S. S. Mycorrhizal plants of traditionally managed boreal grasslands in Norway. *Mycorrhiza* **12**, 117-123 (2002).

106 Likar, M., Bukovnik, U., Kreft, I., Chrungoo, N. K. & Regvar, M. Mycorrhizal status and diversity of fungal endophytes in roots of common buckwheat (Fagopyrum esculentum) and tartary buckwheat (F. tataricum). *Mycorrhiza* **18**, 309-315 (2008).

107 Hayward, J. & Horton, T. R. Phylogenetic trait conservation in the partner choice of a group of ectomycorrhizal trees. *Mol Ecol* **23**, 4886-4898 (2014).

108 Kottke, I., Beck, A., Oberwinkler, F., Homeier, J. & Neill, D. Arbuscular endomycorrhizas are dominant in the organic soil of a neotropical montane cloud forest. *Journal of Tropical Ecology* **20**, 125-129 (2004).

109 Li-Si, Z. & Shun-Xing, G. Molecular diversity of arbuscular mycorrhizal fungi in wild and cultured Gynostemma pentaphyllum roots in Xishuangbanna, Southwest China. *Chinese Journal of Applied Ecology* **24** (2013).

110 Sharma, D. & Kothamasi, D. AMF of mangroves (Genbank accession: KF555269; KF871027; KF871028; KF871030; KF871032; KF555266; KF555271; KF871044). (2013).

111 Selosse, M. A. *et al.* Sebacinales are common mycorrhizal associates of Ericaceae. *New Phytol* **174**, 864-878 (2007).

112 Torrecillas, E., Alguacil, M. M. & Roldán, A. Host preferences of arbuscular mycorrhizal fungi colonizing annual herbaceous plant species in semiarid mediterranean prairies. *Appl Environ Microb* **78**, 6180-6186 (2012).

113 Kjøller, R., Olsrud, M. & Michelsen, A. Co-existing ericaceous plant species in a subarctic mire community share fungal root endophytes. *Fungal Ecology* **3**, 205-214 (2010).

114 Lancellotti, E., Iotti, M., Zambonelli, A. & Franceschini, A. Characterization of Tuber borchii and Arbutus unedo mycorrhizas. *Mycorrhiza* **24**, 481-486 (2014).

115 Setaro, S. D. & Kron, K. Neotropical and north american vaccinioideae (ericaceae) share their mycorrhizal sebacinales-an indication for concerted migration? *PLoS Currents*, 1-19 (2011).

116 Krpata, D. *et al.* High diversity of ectomycorrhizal fungi associated with Arctostaphylos uva-ursi in subalpine and alpine zones: Potential inoculum for afforestation. *Forest Ecology and Management* **250**, 167-175 (2007).

117 Horn, K., Franke, T., Unterseher, M., Schnittler, M. & Beenken, L. Morphological and molecular analyses of fungal endophytes of achlorophyllous gametophytes of Diphasiastrum alpinum (Lycopodiaceae). *American Journal of Botany* **100**, 2158-2174 (2013).

118 Borriello, R. *et al.* Edaphic factors trigger diverse AM fungal communities associated to exotic camellias in closely located Lake Maggiore (Italy) sites. *Mycorrhiza* **25**, 253-265 (2015).

119 Tedersoo, L., Pellet, P., Kõljalg, U. & Selosse, M. A. Parallel evolutionary paths to mycoheterotrophy in understorey Ericaceae and Orchidaceae: Ecological evidence for mixotrophy in Pyroleae. *Oecologia* **151**, 206-217 (2007).

120 Zimmer, K. *et al.* Wide geographical and ecological distribution of nitrogen and carbon gains from fungi in pyroloids and monotropoids (Ericaceae) and in orchids. *New Phytol* **175**, 166-175 (2007).

121 Hutchison, L. J. & Piche, Y. Effects Of Exogenous Glucose On Mycorrhizal Colonization In-Vitro By Early-Stage And Late-Stage Ectomycorrhizal Fungi. *Canadian Journal Of Botany* **73**, 898-904 (1995).

122 Kühdorf, K., Münzenberger, B., Begerow, D., Gómez-Laurito, J. & Hüttl, R. F. Arbutoid mycorrhizas of the genus Cortinarius from Costa Rica. *Mycorrhiza*, 1-17 (2016).

123 Obase, K., Matsuda, Y. & Ito, S. i. Enkianthus campanulatus (Ericaceae) is commonly associated with arbuscular mycorrhizal fungi. *Mycorrhiza* **23**, 199-208 (2013).

124 Grunewaldt-Stöcker, G., Berg, C. v. d., Knopp, J. & Alten, H. v. Interactions of ericoid mycorrhizal fungi and root pathogens in Rhododendron: *In vitro* tests with plantlets in sterile liquid culture. *Plant Root* **7**, 33-48 (2013).

125 Duclos, J. L., Pepin, R. & Bruchet, G. Étude morphologique, anatomique et ultrastructurale d'endomycorhizes synthétiques d' Erica carnea. *Canadian Journal of Botany* **61**, 466-475 (1982).

126 Allen, T. R., Millar, T., Berch, S. M. & Berbee, M. L. Culturing and direct DNA extraction find different fungi from the same ericoid mycorrhizal roots. *New Phytol* **160**, 255-272 (2003).

127 Haug, I. *et al.* Species-rich but distinct arbuscular mycorrhizal communities in reforestation plots on degraded pastures and in neighboring pristine tropical mountain rain forest. *Tropical Ecology* **51**, 125-148 (2010).

128 Vincenot, L. *et al.* Fungal associates of Pyrola rotundifolia, a mixotrophic Ericaceae, from two Estonian boreal forests. *Mycorrhiza* **19**, 15-25 (2008).

129 Tian, W., Zhang, C. Q., Qiao, P. & Milne, R. Diversity of culturable ericoid mycorrhizal fungi of Rhododendron decorum in Yunnan, China. *Mycologia* **103**, 703-709 (2011).

130 Zhang, C., Yin, L. & Dai, S. Diversity of root-associated fungal endophytes in Rhododendron fortunei in subtropical forests of China. *Mycorrhiza* **19**, 417-423 (2009).

131 Harmaja, H. Taxonomic notes on Rhododendron subsection Ledum (Ledum, Ericaceae), with a key to its species. *Annales Botanici Fennici* **28**, 171-173 (1991).

132 Okuda, A., Yamato, M. & Iwase, K. The mycorrhiza of Schizocodon soldanelloides var. magnus (Diapensiaceae) is regarded as ericoid mycorrhiza from its structure and fungal identities. *Mycoscience* **52**, 425-430 (2011).

133 Midgley, D. J., Chambers, S. M. & Cairney, J. W. G. Inorganic and organic substrates as sources of nitrogen and phosphorus for multiple genotypes of two ericoid mycorrhizal fungal taxa from Woollsia pungens and Leucopogon parviflorus (Ericaceae). *Australian Journal of Botany* **52**, 63-71 (2004).

134 Chambers, S. M., Curlevski, N. J. A. & Cairney, J. W. G. Ericoid mycorrhizal fungi are common root inhabitants of non-Ericaceae plants in a south-eastern Australian sclerophyll forest. *FEMS Microbiology Ecology* **65**, 263-270 (2008).

135 Aggangan, N. S., Moon, H. K. & Han, S. H. Growth response of Acacia mangium Willd. seedlings to arbuscular mycorrhizal fungi and four isolates of the ectomycorrhizal fungus Pisolithus tinctorius (Pers.) Coker and Couch. *New Forests* **39**, 215-230 (2010).

136 Weber, J. *et al.* Co-inoculation of Acacia mangium with Glomus intraradices and Bradyrhizobium sp. in aeroponic culture. *Biology and Fertility of Soils* **41**, 233-239 (2005).

137 Diédhiou, A. G. *et al.* The early-stage ectomycorrhizal Thelephoroid fungal sp. is competitive and effective on Afzelia africana Sm. in nursery conditions in Senegal. *Mycorrhiza* **14**, 313-322 (2004).

138 Manoharan, P. T., Pandi, M., Shanmugaiah, V., Gomathinayagam, S. & Balasubramanian, N. Effect of vesicular arbuscular mycorrhizal fungus on the physiological and biochemical changes of five different tree seedlings grown under nursery conditions. *J Biotechnol* **7**, 3431-3436 (2008).

139 Tedersoo, L., Suvi, T., Beaver, K. & Kõljalg, U. Ectomycorrhizal fungi of the Seychelles: Diversity patterns and host shifts from the native Vateriopsis seychellarum (Dipterocarpaceae) and Intsia bijuga (Caesalpiniaceae) to the introduced Eucalyptus robusta (Myrtaceae), but not Pinus caribea (Pinaceae). *New Phytol* **175**, 321-333 (2007).

140 Zhang, Q. *et al.* Positive feedback between mycorrhizal fungi and plants influences plant invasion success and resistance to invasion. *Plos One* **5** (2010).

141 Öpik, M. *et al.* Global sampling of plant roots expands the described molecular diversity of arbuscular mycorrhizal fungi. *Mycorrhiza* **23**, 411-430 (2013).

142 Titus, J. H., Moral, R. d. & Gamiet, S. The distribution of vesicular-arbuscular mycorrhizae on mount St. Helens, Washington. *Madroño* **45**, 162-170 (1998).

143 Rath, M., Weber, H. C. & Imhof, S. Morpho-anatomical and molecular characterization of the mycorrhizas of European Polygala species. *Plant Biology* **15**, 548-557 (2013).

144 Bratek, Z., Jakucs, E., Bóka, K. & Szedlay, G. Mycorrhizae between black locust (Robinia pseudoacacia) and Terfezia terfezioides. *Mycorrhiza* **6**, 271-274 (1996).

145 Ndoye, F. *et al.* Changes in Land Use System and Environmental Factors Affect Arbuscular Mycorrhizal Fungal Density and Diversity, and Enzyme Activities in Rhizospheric Soils of Acacia senegal (L.) Willd. *ISRN Ecology* **2012**, 1-13 (2012).

146 Börstler, B., Renker, C., Kahmen, A. & Buscot, F. Species composition of arbuscular mycorrhizal fungi in two mountain meadows with differing management types and levels of plant biodiversity. *Biology and Fertility of Soils* **42**, 286-298 (2006).

147 Johnson, J.-M. *et al.* Colonization and molecular diversity of arbuscular mycorrhizal fungi associated with the rhizosphere of cowpea (Vigna unguiculata (L.) Walp.) in Benin (West Africa): an exploratory study. *Annals of Microbiology* **66**, 207-221 (2016).

148 Pritsch, K. *et al.* Description and identification of Alnus acuminata ectomycorrhizae from Argentinean alder stands. *Mycologia* **102**, 1263-1273 (2010).

149 Moreau, P.-A. P. A. *et al.* Taxonomy of Alnus-associated hypogeous species of Alpova and Melanogaster (Basidiomycota, Paxillaceae) in Europe. *Cryptogamie Mycologie* **32**, 33-62 (2011).

150 Jargeat, P., Moreau, P.-A., Gryta, H., Chaumeton, J.-P. & Gardes, M. Paxillus rubicundulus (Boletales, Paxillaceae) and two new alder-specific ectomycorrhizal species, Paxillus olivellus and Paxillus adelphus, from Europe and North Africa. *Fungal Biol-Uk* **120**, 711-728 (2016).

151 Tedersoo, L., Suvi, T., Jairus, T., Ostonen, I. & Põlme, S. Revisiting ectomycorrhizal fungi of the genus Alnus: Differential host specificity, diversity and determinants of the fungal community. *New Phytol* **182**, 727-735 (2009).

152 Sýkorová, Z. *et al.* Forest reclamation of fly ash deposit: A field study on appraisal of mycorrhizal inoculation. *Restoration Ecology* **24**, 184-193 (2016).

153 Chatarpaul, L., Chakravarty, P. & Subramaniam, P. Studies in tetrapartite symbioses - I. Role of ecto- and endomycorrhizal fungi and Frankia on the growth performance of Alnus incana. *Plant Soil* **118**, 145-150 (1989).

154 Ilyas, S., Razaq, A. & Khalid, A. N. *Inocybe nitidiuscula* and its ectomycorrhizae associated with *Alnus nitida* from Galyat, Pakistan. *Mycotaxon* **124**, 247-254 (2013).

155 Ashraf, T., Hanif, M. & Khalid, A. N. *Peziza michelii* and its ectomycorrhizae with *Alnus nitida* (Betulaceae) from Pakistan. *Mycotaxon* **120**, 181-188 (2012).

156 Kennedy, P. G. & Hill, L. T. A molecular and phylogenetic analysis of the structure and specificity of Alnus rubra ectomycorrhizal assemblages. *Fungal Ecology* **3**, 195-204 (2010).

157 Coburn, J. *The effects of selective cutting and liming on mushroom formation and mycorrhizal colonization of mature yellow birch (Betula alleghaniensis Britt.) in a deciduous forest within the Reserve faunique de Portneuf, south central Quebec*, (2003).

158 Poznanovic, S. K., Lilleskov, E. A. & Webster, C. R. Sharing rotting wood in the shade: ectomycorrhizal communities of co-occurringbirch and hemlock seedlings. *Mycorrhiza* **25**, 153-164 (2014).

159 Twieg, B. D., Durall, D. M., Simard, S. W. & Jones, M. D. Influence of soil nutrients on ectomycorrhizal communities in a chronosequence of mixed temperate forests. *Mycorrhiza* **19**, 305-316 (2009).

160 Pasonen, H. L. *et al.* Effects of sugar beet chitinase IV on root-associated fungal community of transgenic silver birch in a field trial. *Planta* **230**, 973-983 (2009).

161 Bidartondo, M. I., Bruns, T. D., Weiss, M., Sérgio, C. & Read, D. J. Specialized cheating of the ectomycorrhizal symbiosis by an epiparasitic liverwort. *Proceedings of the Royal Society B: Biological Sciences* **270**, 835-842 (2003).

162 Hashimoto, Y. & Hyakumachi, M. Quantities and types of ectomycorrhizal and endophytic fungi associated with Betula platyphylla var. japonica seedlings during the initial stage of establishment of vegetation after disturbance. *Ecological Research* **15**, 21-31 (2000).

163 Vrålstad, T., Myhre, E. & Schumacher, T. Molecular diversity and phylogenetic affinities of symbiotic root-associated ascomycetes of the Helotiales in burnt and metal polluted habitats. *New Phytol* **155**, 131-148 (2002).

164 Bonito, G., Brenneman, T. & Vilgalys, R. Ectomycorrhizal fungal diversity in orchards of cultivated pecan (Carya illinoinensis; Juglandaceae). *Mycorrhiza* **21**, 601-612 (2011).

165 Bauman, J. M., Keiffer, C. H., Hiremath, S. & McCarthy, B. C. Soil preparation methods promoting ectomycorrhizal colonization and American chestnut Castanea dentata establishment in coal mine restoration. *Journal of Applied Ecology* **50**, 721-729 (2013).

166 D'Amico, K. M. *et al.* Comparisons of Ectomycorrhizal Colonization of Transgenic American Chestnut with Those of the Wild Type, a Conventionally Bred Hybrid, and Related Fagaceae Species. *Appl Environ Microb* **81**, 100-108 (2015).

167 Chen, S. *et al.* Ectomycorrhizae symbiosis in Castanea mollissima improves phosphate acquisition through activating gene expression and H+ efflux. *Scientia Horticulturae* **210**, 99-107 (2016).

168 Wang, Q., Gao, C. & Guo, L. D. Ectomycorrhizae associated with Castanopsis fargesii (Fagaceae) in a subtropical forest, China. *Mycol Prog* **10**, 323-332 (2011).

169 Theodorou, C. & Reddell, P. Invitro Synthesis of Ectomycorrhizas on Casuarinaceae With a Range of Mycorrhizal Fungi. *New Phytol* **118**, 279-288 (1991).

170 Sempavalan, J., Wheeler, C. T. & Hooker, J. E. Lack of competition between Frankia and Glomus for infection and colonization of roots of Casuarina equisetifolia (L.). *New Phytol* **130**, 429-436 (1995).

171 Benucci, G. M. N. *et al.* Ectomycorrhizal communities in a productive Tuber aestivum Vittad. orchard: Composition, host influence and species replacement. *FEMS Microbiology Ecology* **76**, 170-184 (2011).

172 Montoya, L., Haug, I. & Bandala, V. M. Two Lactarius species associated with a relict Fagus grandifolia var. mexicana population in a Mexican montane cloud forest. *Mycologia* **102**, 153-162 (2010).

173 Garay-Serrano, E., Bandala, V. M. & Montoya, L. Morphological and molecular identification of the ectomycorrhizal association of Lactarius fumosibrunneus and Fagus grandifolia var. mexicana trees in eastern Mexico. *Mycorrhiza* **22**, 583-588 (2012).

174 Trocha, L. K., Weiser, E. & Robakowski, P. Interactive effects of juvenile defoliation, light conditions, and interspecific competition on growth and ectomycorrhizal colonization of Fagus sylvatica and Pinus sylvestris seedlings. *Mycorrhiza* **26**, 47-56 (2016).

175 Erős-Honti, Z., Kovács, G. M., Szedlay, G. & Jakucs, E. Morphological and molecular characterization of Humaria and Genea ectomycorrhizae from Hungarian deciduous forests. *Mycorrhiza* **18**, 133-143 (2008).

176 Ilyas, S., Razaq, A. & Khalid, A. N. Molecular Investigations to Determine the Ectomycorrhizal Habit of Lactarius sanguifluus Associated with Coniferous and Deciduous Vegetation of Galyat, Khyber Pakhtunkhwa, Pakistan. *International Journal of Agriculture & Biology* **15**, 857-863 (2013).

177 Dolcet-Sanjuan, R., Claveria, E., Camprubí, A., Estaún, V. & Calvet, C. Micropropagation of walnut trees (Juglans regia L) and response to arbuscular mycorrhizal inoculation. *Agronomie* **16**, 639-645 (1996).

178 Tedersoo, L. *et al.* Establishment of ectomycorrhizal fungal community on isolated Nothofagus cunninghamii seedlings regenerating on dead wood in Australian wet temperate forests: Does fruit-body type matter? *Mycorrhiza* **19**, 403-416 (2009).

179 Orlovich, D. a., Draffin, S. J., Daly, R. a. & Stephenson, S. L. Piracy in the high trees: ectomycorrhizal fungi from an aerial 'canopy soil' microhabitat. *Mycologia* **105**, 52-60 (2013).

180 Egerton-Warburton, L. & Allen, M. F. Endo- and ectomycorrhizas in Quercus agrifolia Nee. (Fagaceae): Patterns of root colonization and effects on seedling growth. *Mycorrhiza* **11**, 283-290 (2001).

181 Southworth, D. (eds Cládio Aleixo Chuteira & Abrahan Bispo Grao) 207-218 (Nova Science Publishers, 2013).

182 Pereira, G. *et al.* Using common mycorrhizal networks for controlled inoculation of Quercus spp. with Tuber melanosporum: The nurse plant method. *Mycorrhiza* **23**, 373-380 (2013).

183 Jakucs, E., Kovács, G. M., Agerer, R., Romsics, C. & Erős-Honti, Z. Morphological-anatomical characterization and molecular identification of Tomentella stuposa ectomycorrhizae and related anatomotypes. *Mycorrhiza* **15**, 247-258 (2005).

184 Huang, J. *et al.* Ectomycorrhizal fungal communities associated with Masson pine (Pinus massoniana) and white oak (Quercus fabri) in a manganese mining region in Hunan Province, China. *Fungal Ecology* **9**, 1-10 (2014).

185 Ilyas, S. *Molecular investigations to characterize ectomycorrhizal fungal communities associated with some decidious trees of Galyat, Pakistan*, (2013).

186 Healy, R. A. *et al.* High diversity and widespread occurrence of mitotic spore mats in ectomycorrhizal Pezizales. *Mol Ecol* **22**, 1717-1732 (2012).

187 Bonito, G. *et al.* Plant host and soil origin influence fungal and bacterial assemblages in the roots of woody plants. *Mol Ecol* **23**, 3356-3370 (2014).

188 Giomaro, G. *et al.* Comparative study and molecular characterization of ectomycorrhizas in Tilia americana and Quercus pubescens with Tuber brumale. *Fems Microbiol Lett* **216**, 9-14 (2002).

189 Trocha, L. K. *et al.* Ectomycorrhizal fungal communities of native and non-native Pinus and Quercus species in a common garden of 35-year-old trees. *Mycorrhiza* **22**, 121-134 (2012).

190 Healy, R. A., Zurier, H., Bonito, G., Smith, M. E. & Pfister, D. H. Mycorrhizal detection of native and non-native truffles in a historic arboretum and the discovery of a new North American species, Tuber arnoldianum sp. nov. *Mycorrhiza*, 1-12 (2016).

191 Dickie, I. A., Koide, R. T. & Fayish, A. C. Vesicular-arbuscular mycorrhizal infection of Quercus rubra seedlings. *New Phytol* **151**, 257-264 (2001).

192 Kayama, M. & Yamanaka, T. Growth characteristics of ectomycorrhizal seedlings of Quercus glauca, Quercus salicina, and Castanopsis cuspidata planted on acidic soil. *Trees - Structure and Function* **28**, 569-583 (2014).

193 Lancellotti, E. & Franceschini, A. Studies on the ectomycorrhizal community in a declining Quercus suber L. stand. *Mycorrhiza* **23**, 533-542 (2013).

194 Zong, K. *et al.* Inoculation of ectomycorrhizal fungi contributes to the survival of tree seedlings in a copper mine tailing. *Journal of Forest Research* **20**, 493-500 (2015).

195 Heenan, P. B. & Smissen, R. D. Revised circumscription of Nothofagus and recognition of the segregate genera Fuscospora, Lophozonia, and Trisyngyne (Nothofagaceae). *Phytotaxa* **146**, 1-1 (2013).

196 Prin, Y. *et al.* in *The Mycorrhizal Symbiosis in Mediterranean Environment: Importance in Ecosystem Stability and in Soil Rehabilitation Strategies* (2012).

197 De Beenhouwer, M. *et al.* Changing soil characteristics alter the arbuscular mycorrhizal fungi communities of Arabica coffee (Coffea arabica) in Ethiopia across a management intensity gradient. *Soil Biology and Biochemistry* **91**, 133-139 (2015).

198 Day, N. J., Antunes, P. M. & Dunfield, K. E. Changes in arbuscular mycorrhizal fungal communities during invasion by an exotic invasive plant. *Acta Oecologica* **67**, 66-74 (2015).

199 Gupta, N., Basak, U. C. & Das, P. Arbuscular mycorrhizal association of mangroves in saline and non-saline soils. *Mycorrhiza News* **13**, 14-19 (2002).

200 D'Souza, J. & Rodrigues, B. F. Biodiversity of Arbuscular Mycorrhizal (AM) fungi in mangroves of Goa in West India. *Journal of Forestry Research* **24**, 515-523 (2013).

201 Nalian, A., van Kley, J., Stroup, K. & Martynova -Van Kley, A. Host Preferences of Arbuscular Mycorrhizae Along a Soil Nutrient and Hydrological Gradient. 76-81 (2010).

202 Kohout, P. *et al.* Comparison of commonly used primer sets for evaluating arbuscular mycorrhizal fungal communities: Is there a universal solution? *Soil Biology and Biochemistry* **68**, 482-493 (2014).

203 Sýkorová, Z., Ineichen, K., Wiemken, A. & Redecker, D. The cultivation bias: Different communities of arbuscular mycorrhizal fungi detected in roots from the field, from bait plants transplanted to the field, and from a greenhouse trap experiment. *Mycorrhiza* **18**, 1-14 (2007).

204 Long, L. K. *et al.* Molecular community analysis of arbuscular mycorrhizal fungi associated with five selected plant species from heavy metal polluted soils. *European Journal of Soil Biology* **46**, 288-294 (2010).

205 Yoshimura, Y., Ido, A., Iwase, K., Matsumoto, T. & Yamato, M. Communities of Arbuscular Mycorrhizal Fungi in the Roots of Pyrus pyrifolia var. culta (Japanese Pear) in Orchards with Variable Amounts of Soil-Available Phosphorus. *Microbes and Environments* **28**, 105-111 (2013).

206 Staddon, P. L., Graves, J. D. & Fitter, A. H. Effect of enhanced atmospheric CO2 on mycorrhizal colonization and phosphorus inflow in 10 herbaceous species of contrasting growth strategies. *Funct Ecol* **13**, 190-199 (1999).

207 Chaiyasen, A., Young, J. P. W., Teaumroong, N., Gavinlertvatana, P. & Lumyong, S. Characterization of arbuscular mycorrhizal fungus communities of aquilaria crassna and tectona grandis roots and soils in thailand plantations. *Plos One* **9** (2014).

208 Bedini, S., Turrini, A., Rigo, C., Argese, E. & Giovannetti, M. Molecular characterization and glomalin production of arbuscular mycorrhizal fungi colonizing a heavy metal polluted ash disposal island, downtown Venice. *Soil Biology and Biochemistry* **42**, 758-765 (2010).

209 Siri-in, J., Kumla, J., Suwannarach, N. & Lumyong, S. Culture Conditions and Some Properties of Pure Culture of Ectomycorrhizal Fungus , Scleroderma sinnamariense. *Chiang Mai Journal of Science* **41**, 275-285 (2014).

210 Kumar, T., Majumdar, a., Das, P., Sarafis, V. & Ghose, M. Trypan blue as a fluorochrome for confocal laser scanning microscopy of arbuscular mycorrhizae in three mangroves. *Biotechnic & Histochemistry* **83**, 153-159 (2008).

211 Sharma, D. & Kothamasi, D. Arbuscular mycorrhizal fungal (AMF) diversity in mangroves (Genbank accession: KF871063; KF871057; KF871056; KF555276; KF555268). (2014).

212 Shepherd, M., Nguyen, L., Jones, M. E., Nichols, J. D. & Carpenter, F. L. A method for assessing arbuscular mycorrhizal fungi group distribution in tree roots by intergenic transcribed sequence variation. *Plant Soil* **290**, 259-268 (2007).

213 Sharma, D. & Kothamasi, D. (2014).

214 Davison, J. *et al.* Global assessment of arbuscular mycorrhizal fungus diversity reveals very lowendemism. *Science* **127**, 970-973 (2015).

215 Solaiman, Z. M. & Abbott, L. K. Influence of arbuscular mycorrhizal fungi, inoculum level and phosphorus placement on growth and phosphorus uptake of Phyllanthus calycinus under jarrah forest soil. *Biology and Fertility of Soils* **44**, 815-821 (2008).

216 Kovács, G. M. & Jakucs, E. Morphological and molecular comparison of white truffle ectomycorrhizae. *Mycorrhiza* **16**, 567-574 (2006).

217 Cicatelli, A. *et al.* Arbuscular mycorrhizal fungi restore normal growth in a white poplar clone grown on heavy metal-contaminated soil, and this is associated with upregulation of foliar metallothionein and polyamine biosynthetic gene expression. *Annals of Botany* **106**, 791-802 (2010).

218 Grubisha, L. C., Levsen, N., Olson, M. S. & Lee Taylor, D. Intercontinental divergence in the Populus-associated ectomycorrhizal fungus, Tricholoma populinum. *New Phytol* **194**, 548-560 (2012).

219 Meng, X.-J. & Tang, F.-R. Study on the promotion effects of mycorrhizae to Populus davidiana seedlings. *Mycosystema* **4** (2001).

220 Chen, L. *et al.* Sex-specific responses of Populus deltoides to Glomus intraradices colonization and Cd pollution. *Chemosphere* **155**, 196-206 (2016).

221 Wang, Y. S., Chen, L., Zhang, S. B. & Liu, Y. Y. Biodiversity of arbuscular mycorrhizal fungi in the natural forests of Populus euphratica and Alhagi sparsifolia in Xinjiang. *Arid Zone Research* **27**, 927-932 (2010).

222 Gryta, H., Carriconde, F., Charcosset, J. Y., Jargeat, P. & Gardes, M. Population dynamics of the ectomycorrhizal fungal species Tricholoma populinum and Tricholoma scalpturatum associated with black poplar under differing environmental conditions. *Environ Microbiol* **8**, 773-786 (2006).

223 Long, D., Liu, J., Han, Q., Wang, X. & Huang, J. Ectomycorrhizal fungal communities associated with Populus simonii and Pinus tabuliformis in the hilly-gully region of the Loess Plateau, China. *Scientific Reports* **6**, 24336-24336 (2016).

224 Tedersoo, L., Hansen, K., Perry, B. A. & Kjøller, R. Molecular and morphological diversity of pezizalean ectomycorrhiza. *New Phytol* **170**, 581-596 (2006).

225 Tedersoo, L. & Põlme, S. Infrageneric variation in partner specificity: Multiple ectomycorrhizal symbionts associate with Gnetum gnemon (Gnetophyta) in Papua New Guinea. *Mycorrhiza* **22**, 663-668 (2012).

226 Martin, F. *et al.* Symbiotic sequencing for the Populus mesocosm. *New Phytol* **161**, 330-335 (2004).

227 Martin, F. *et al.* The long hard road to a completed Glomus intraradices genome. *New Phytol* **180**, 747-750 (2008).

228 Sumorok, B. & Kiedrzynska, E. Mycorrhizal status of native willow species in the Pilica River floodplain along the moisture gradient. *Wetlands: Monitoring, Modelling and Management*, 281-286 ST - Mycorrhizal status of native willow (2007).

229 Timling, I. *et al.* Distribution and drivers of ectomycorrhizal fungal communities across the North American Arctic. *Ecosphere* **3**, 1-25 (2012).

230 Mühlmann, O. & Peintner, U. Mycobionts of Salix herbacea on a glacier forefront in the Austrian Alps. *Mycorrhiza* **18**, 171-180 (2008).

231 Hrynkiewicz, K., Baum, C. & Leinweber, P. Mycorrhizal community structure, microbial biomass P and phosphatase activities under Salix polaris as influenced by nutrient availability. *European Journal of Soil Biology* **45**, 168-175 (2009).

232 Wu, B., Nara, K. & Hogetsu, T. Genetic structure of Cenococcum geophilum populations in primary successional volcanic deserts on Mount Fuji as revealed by microsatellite markers. *New Phytol* **165**, 285-293 (2005).

233 Nara, K. Ectomycorrhizal networks and seedling establishment during early primary succession. *New Phytol* **169**, 169-178 (2006).

234 Ryberg, M., Larsson, E. & Molau, U. Ectomycorrhizal Diversity on Dryas octopetala and Salix reticulata in an Alpine Cliff Ecosystem. *Arctic, Antarctic, and Alpine Research* **41**, 506-514 (2009).

235 Yuwa-Amornpitak, T., Vichitsoonthonkul, T., Tanticharoen, M., Cheevadhanarak, S. & Ratchadawong, S. Diversity of ectomycorrhizal fungi on Dipterocarpaceae in Thailand. *Journal of Biological Sciences* **6**, 1059-1064 (2006).

236 Lang, C., Seven, J. & Polle, A. Host preferences and differential contributions of deciduous tree species shape mycorrhizal species richness in a mixed Central European forest. *Mycorrhiza* **21**, 297-308 (2011).

237 Harley, J. L. & Harley, E. L. A check-list of mycorrhizae in the british flora. *New Phytologist* **105**, 1-102 (1987).

238 Fini, A. *et al.* Effect of controlled inoculation with specific mycorrhizal fungi from the urban environment on growth and physiology of containerized shade tree species growing under different water regimes. *Mycorrhiza* **21**, 703-719 (2011).

239 Martin, F., Diez, J., Dell, B. & Delaruelle, C. Phylogeography of the ectomycorrhizal Pisolithus species as inferred from nuclear ribosomal DNA ITS sequences. *New Phytol* **153**, 345-357 (2002).

240 Pennington, H. G., Bidartondo, M. I. & Barsoum, N. A few exotic mycorrhizal fungi dominate eucalypts planted in England. *Fungal Ecology* **4**, 299-302 (2011).

241 Glen, M., Tommerup, I. C., Bougher, N. L. & O'Brien, P. A. Are Sebacinaceae common and widespread ectomycorrhizal associates of Eucalyptus species in Australian forests? *Mycorrhiza* **12**, 243-247 (2002).

242 Sharma, D. & Kothamasi, D. Arbuscular mycorrhizal fungal (AMF) diversity in mangroves II (Genbank accession: KF871010, KF871009, KF871004, KF555259, KF871000, KF871010, KF871009, KF871004, KF555259, KF871000), <http://www.ncbi.nlm.nih.gov/> (2014).

243 Renker, C., Blanke, V. & Buscot, F. Diversity of arbuscular mycorrhizal fungi in grassland spontaneously developed on area polluted by a fertilizer plant. *Environ Pollut* **135**, 255-266 (2005).

244 Yamato, M., Ogura-Tsujita, Y., Takahashi, H. & Yukawa, T. Significant difference in mycorrhizal specificity between an autotrophic and its sister mycoheterotrophic plant species of Petrosaviaceae. *Journal of Plant Research* **127**, 685-693 (2014).

245 Kandiannan, K., Sivaraman, K., Anandaraj, M. & Krishnamurthy, K. S. Growth and nutrient content of black pepper (Piper nigrum L.) cuttings as influenced by inoculation with biofertilizers. *Journal of Spices and Aromatic Crops* **9**, 145-147 (2000).

246 Busby, R. R., Stromberger, M. E., Rodriguez, G., Gebhart, D. L. & Paschke, M. W. Arbuscular mycorrhizal fungal community differs between a coexisting native shrub and introduced annual grass. *Mycorrhiza* **23**, 129-141 (2013).

247 Mühlmann, O. & Peintner, U. Ectomycorrhiza of Kobresia myosuroides at a primary successional glacier forefront. *Mycorrhiza* **18**, 355-362 (2008).

248 Kawahara, A. & Ezawa, T. Characterization of arbuscular mycorrhizal fungal communities with respect to zonal vegetation in a coastal dune ecosystem. *Oecologia* **173**, 533-543 (2013).

249 Rajeshkannan, V., Sumathi, C. S. & Manian, S. Arbuscular Mycorrhizal Fungi Colonization in Upland Rice as Influenced by Agrochemical Application. *Rice Science* **16**, 307-313 (2009).

250 Moore, V. C., Dzantor, E. K. & Hui, D. Mycorrhizal Enhancement of Biomass Productivity of Big Bluestem and Switchgrass in Neutral and Acidic Substrate. *Journal of Applied Biosciences* **89**, 8263-8271 (2015).

251 Tomeo, N. J. & Springer, C. J. *Mycorrhizal communities in a precipitation variability experiment (Genbank accession: JX276924 JX276923 JX276922 JX276921 JX276920)*, <http://www.ncbi.nlm.nih.gov/> (2012).

252 Sýkorová, Z. *et al.* Long-term tracing of Rhizophagus irregularis isolate BEG140 inoculated on Phalaris arundinacea in a coal mine spoil bank, using mitochondrial large subunit rDNA markers. *Mycorrhiza* **22**, 69-80 (2012).

253 Wilde, P. *et al.* Biodiversity of arbuscular mycorrhizal fungi in roots and soils of two salt marshes. *Environ Microbiol* **11**, 1548-1561 (2009).

254 Tang, F., White, J. A. & Charvat, I. The effect of phosphorus availability on arbuscular mycorrhizal colonization of Typha angustifolia. *Mycologia* **93**, 1042-1047 (2001).

255 Ray, A. M. & Inouye, R. S. Effects of water-level fluctuations on the arbuscular mycorrhizal colonization of Typha latifolia L. *Aquatic Botany* **84**, 210-216 (2006).

256 Calheiros, C., Franco, A. & Castro, P. Study on the dynamics of microbial communities in a constructed wetland inhabited by different plant species with focus on arbucular mycorrhizal fungi (Genbank accession: KJ639003 KJ639005 KJ639006 KJ639008 KJ639009), <http://www.ncbi.nlm.nih.gov/> (2014).

257 Moora, M., Öpik, M., Sen, R. & Zobel, M. Native arbuscular mycorrhizal fungal communities differentially influence the seedling performance of rare and common Pulsatilla species. *Funct Ecol* **18**, 554-562 (2004).

258 Öpik, M. *et al.* Divergent arbuscular mycorrhizal fungal communities colonize roots of Pulsatilla spp. in boreal Scots pine forest and grassland soils. *New Phytol* **160**, 581-593 (2003).

259 Kołaczek, P., Zubek, S., Błaszkowski, J., Mleczko, P. & Margielewski, W. Erosion or plant succession - How to interpret the presence of arbuscular mycorrhizal fungi (Glomeromycota) spores in pollen profiles collected from mires. *Review of Palaeobotany and Palynology* **189**, 29-37 (2013).

260 Krüger, M., Stockinger, H., Krüger, C. & Schüssler, A. DNA-based species level detection of Glomeromycota: one PCR primer set for all arbuscular mycorrhizal fungi. *The New phytologist* **183**, 212-223 (2009).

261 Ryszka, P., Zarzyka-Ryszka, M., T., A., Maciej, C. & Turnau, K. Arbuscular mycorrhizal fungi from petroleum-impacted sites in the Polish Carpathians (Genbank accession: KP284252; KP284251; KP284250; KP284249; KP284248-KP284252; KP284251; KP284250; KP284249; KP284248), <www.ncbi.nlm.nih.gov> (2015).

262 McDonald, K. R., Pennell, J., Frank, J. L. & Southworth, D. Ectomycorrhizas of Cercocarpus ledifolius (rosaceae). *American Journal of Botany* **97**, 1867-1872 (2010).

263 Runjin, L. Effects of vesicular-arbuscular mycorrhizas and phosphorus on water status and growth of Malus hupehensis. *Journal of Plant Nutrition* **12**, 997-1017 (1989).

264 An, Z. Q., Shen, T. & Wang, H. G. Mycorrhizal fungi in relation to growth and mineral nutrition of apple seedlings. *Scientia Horticulturae* **54**, 275-285 (1993).

265 Yoh-ichi Matsubara, T. & Karikomi. Effect of Arbuscular Mycorrhizal Fungus Inoculation on Growth of Apple (Malus ssp .) Seedlings. *Japan. Soc. Hort. Sci* **65**, 297-302 (1996).

266 Palmer, J. M., Lindner, D. L. & Volk, T. J. Ectomycorrhizal characterization of an American chestnut (Castanea dentata)-dominated community in Western Wisconsin. *Mycorrhiza* **19**, 27-36 (2008).

267 Helgason, T., Feng, H., Sherlock, D. J., Young, J. P. W. & Fitter, A. H. Arbuscular mycorrhizal communities associated with maples (Acer spp .) in a common garden are influenced by season and host plant. *Botany* **326**, 321-326 (2014).

268 Pham, N. D. H. *et al.* A sheathing mycorrhiza between the tropical bolete Phlebopus spongiosus and Citrus maxima. *Mycoscience* **53**, 347-353 (2012).

269 Youpensuk, S., Lordkaew, S. & Rerkasem, B. Genotypic Variation in Responses of Citrus spp . to Arbuscular Mycorrhizal Fungi. *Journal of Agriculture Science* **1**, 59-65 (2009).

270 Kumla, J., Bussaban, B., Suwannarach, N., Lumyong, S. & Danell, E. Basidiome formation of an edible wild, putatively ectomycorrhizal fungus, Phlebopus portentosus without host plant. *Mycologia* **104**, 597-603 (2012).

271 Brundett, M., Dell, B., Malajczuk, N. & Mingqi, G. (eds M. Brundett, B. Dell, N. Malajczuk, & Gong Mingqin) 146-146.

272 Baraka, M. A., Ramadan, E.-s. M. & Mohamed, A. F. Biodiversity and Classification of Arbuscular Mycorrhizal Fungi (Glomales) in Ismailia Governorate. *Agricultural Research Journal, Suez Canal University* **12**, 83-90 (2012).

273 Mohandas, S. Arbuscular mycorrhizal fungi benefit mango (Mangifera indica L.) plant growth in the field. *Scientia Horticulturae* **143**, 43-48 (2012).

274 Zhang, H. H., Tang, M., Chen, H., Wang, Y. & Ban, Y. Arbuscular mycorrhizas and dark septate endophytes colonization status in medicinal plant Lycium barbarum L. in arid Northwestern China. *African Journal of Microbiology Research* **4**, 1914-1920 (2010).

275 Zhang, H. & Tang, M. *Arbuscular mycorrhizal community and glomalin content associated with the rhizosphere of Lycium barbarum in arid Northwestern China (Genbank accession: JF432017, JF432016, JF432015, JF432014, JF432013)*, <www.ncbi.nlm.nih.gov> (2011).

276 Cesaro, P. *et al.* Preferential colonization of Solanum tuberosum L. Roots by the fungus Glomus intraradices in arable soil of a potato farming area. *Appl Environ Microb* **74**, 5776-5783 (2008).

277 Koffi, M. C. & Declerck, S. In vitro mycorrhization of banana (Musa acuminata) plantlets improves their growth during acclimatization. *In Vitro Cellular and Developmental Biology - Plant* **51**, 265-273 (2015).

278 Da Silva, M. F., Pescador, R., Rebelo, R. A. & Stürmer, S. L. The effect of arbuscular mycorrhizal fungal isolates on the development and oleoresin production of micropropagated Zingiber officinale. *Brazilian Journal of Plant Physiology* **20**, 119-130 (2008).

279 Gray, N. E. A taxonomic revision of Podocarpus: VII. The African species of Podocarpus: section Afrocarpus. *Journal of the Arnold Arboretum* **34**, 67-76 (1953).

280 Wubet, T., Weiß, M., Kottke, I. & Oberwinkler, F. Two threatened coexisting indigenous conifer species in the dry Afromontane forests of Ethiopia are associated with distinct arbuscular mycorrhizal fungal communities. *Canadian Journal of Botany* **84**, 1617-1627 (2006).

281 Zandavalli, R. B., Dillenburg, L. c. R. & De Souza, P. V. D. Growth responses of Araucaria angustifolia (Araucariaceae) to inoculation with the mycorrhizal fungus Glomus clarum. *Appl Soil Ecol* **25**, 245-255 (2004).

282 Moreira, M., Gomes, J. E., Cardoso, E. J. B. N. & Tsai, S. M. *Diversity of the arbuscular mycorrhizal fungi in tropical ecosystems (Genbank accession: AY919856 AY919855 AY919852 AY919848 AY919846)*, <www.ncbi.nlm.nih.gov> (2005).

283 Kottke, I. & Haug, I. The significance of mycorrhizal diversity of trees in the tropical mountain forest of southern Ecuador. *Lyonia* **7**, 49-56 (2004).

284 Haug, I., Oberwinkler, F. & Kottke, I. *Distinctive communities of Glomeromycota in a neotropical mountain rain forest and a neighboring afforestation site in southern Ecuador (Genbank accession: EU152145; EU152144)*, <www.ncbi.nlm.nih.gov> (2008).

285 Bidartondo, M. I. *et al.* Epiparasitic plants specialized on arbuscular mycorrhizal fungi. *Nature* **419**, 389-392 (2002).

286 Jargeat, P. *et al.* Phylogenetic species delimitation in ectomycorrhizal fungi and implications for barcoding: The case of the Tricholoma scalpturatum complex (Basidiomycota). *Mol Ecol* **19**, 5216-5230 (2010).

287 Fontana, A. Vesicular-Arbuscular Mycorrhizas of Ginkgo-Biloba L in Natural and Controlled Conditions. *New Phytol* **99**, 441-447 (1985).

288 Rudawska, M., Pietras, M., Smutek, I., Strzeliński, P. & Leski, T. Ectomycorrhizal fungal assemblages of Abies alba Mill. outside its native range in Poland. *Mycorrhiza* **26**, 57-65 (2016).

289 Kummel, M. & Lostroh, P. Altering light availability to the plant host determined the identity of the dominant ectomycorrhizal fungal partners and mediated mycorrhizal effects on plant growth. *Botany* **89**, 439-450 (2011).

290 Miyamoto, Y., Nakano, T., Hattori, M. & Nara, K. The mid-domain effect in ectomycorrhizal fungi: range overlap along an elevation gradient on Mount Fuji, Japan. *The ISME Journal* **8**, 1739-1746 (2014).

291 Bidartondo, M. I., Kretzer, A., Pine, E. & Bruns, T. D. High root concentration and uneven ectomycorrhizal diversity near Sarcodes sanguinea (Ericaceae): a cheater that stimulates its victims? *American Journal of Botany* **87**, 1783-1788 (2000).

292 Argüelles-Moyao, A., Garibay-Orijel, R., Márquez-Valdelamar, L. M. & Arellano-Torres, E. Clavulina-Membranomyces is the most important lineage within the highly diverse ectomycorrhizal fungal community of Abies religiosa. *Mycorrhiza*, 1-13 (2016).

293 Lamus, V. *et al.* Mycorrhizal synthesis of the edible mushroom Turbinellus floccosus with Abies religiosa from central Mexico. *Mycoscience* **56**, 622-626 (2015).

294 Hanif, M., Khalid, A. N. & Sarwer, S. Additions to the ectomycorrhizae associated with Himalayan cedar (Cedrus deodara) using rDNA-ITS. *International Journal of Agriculture and Biology* **14**, 101-106 (2012).

295 Dar, G. H., Beig, M. A. & Ganai, N. A. Influence of Ectomycorrhizal Inoculation on Blue Pine (Pinus wallichiania) and Deodar (Cedrus deodara) Seedlings. *Trends in Biosciences* **3**, 60-62 (2010).

296 Ge, Z. W., Smith, M. E., Zhang, Q. Y. & Yang, Z. L. Two species of the Asian endemic genus Keteleeria form ectomycorrhizas with diverse fungal symbionts in southwestern China. *Mycorrhiza* **22**, 403-408 (2012).

297 Leski, T. & Rudawska, M. Ectomycorrhizal fungal community of naturally regenerated European larch (Larix decidua) seedlings. *Symbiosis* **56**, 45-53 (2012).

298 Fan, Y. & Yan, W. *Some of trees root tip ectomycorrhizal fungus sequence from inner Mongolia in China (Genbank accession: FJ803938.1 through FJ803950.1)*, <http://www.ncbi.nlm.nih.gov/> (2010).

299 Menkis, A., Uotila, A., Arhipova, N. & Vasaitis, R. Effects of stump and slash removal on growth and mycorrhization of Picea abies seedlings outplanted on a forest clear-cut. *Mycorrhiza* **20**, 505-509 (2010).

300 Liyuan, F. Y. & Wang, Y. W. Morphological Type and Molecular Identification of Ectomycorrhizae on Picea crassifolia in Helan Mountain. *Scientia Silvae Sinicae* **6**, 018-018 (2011).

301 Kernaghan, G., Sigler, L. & Khasa, D. Mycorrhizal and root endophytic fungi of containerized Picea glauca seedlings assessed by rDNA sequence analysis. *Microbial Ecology* **45**, 128-136 (2003).

302 Reithmeier, L. & Kernaghan, G. Availability of ectomycorrhizal fungi to black spruce above the present treeline in Eastern Labrador. *Plos One* **8**, e77527-e77527 (2013).

303 Wilcox, H. E. & Wang, C. J. K. Ectomycorrhizal and ectendomycorrhizal associations of Phialophora finlandia with Pinus resinosa, Picea rubens, and Betula alleghaniensis. *Canadian Journal of Forest Research* **17**, 976-990 (1987).

304 Carfrae, J. A., Skene, K. R., Sheppard, L. J., Ingleby, K. & Crossley, A. Effects of nitrogen with and without acidified sulphur on an ectomycorrhizal community in a Sitka spruce (Picea sitchensis Bong. Carr) forest. *Environ Pollut* **141**, 131-138 (2006).

305 Niazi, A. R., Iqbal, S. H. & Khalid, A. N. Ectomycorrhizae between Amanita rubescens and Himalayan spruce (Picea smithiana) from Pakistan. *Mycotaxon* **107**, 73-80 (2009).

306 Mohatt, K. R., Cripps, C. L. & Lavin, M. Ectomycorrhizal fungi of whitebark pine (a tree in peril) revealed by sporocarps and molecular analysis of mycorrhizae from treeline forests in the Greater Yellowstone Ecosystem. *Botany* **86**, 14-25 (2008).

307 LeDuc, S. D., Lilleskov, E. A., Horton, T. R. & Rothstein, D. E. Ectomycorrhizal fungal succession coincides with shifts in organic nitrogen availability and canopy closure in post-wildfire jack pine forests. *Oecologia* **172**, 257-269 (2013).

308 Hortal, S., Pera, J., Galipienso, L. & Parladé, J. Molecular identification of the edible ectomycorrhizal fungus Lactarius deliciosus in the symbiotic and extraradical mycelium stages. *J Biotechnol* **126**, 123-134 (2006).

309 Rivera, Y. & Horton, T. R. Exotic ectomycorrhizal fungal communities associated with *Pinus caribaea* on the island of Puerto Rico, <http://www.ncbi.nlm.nih.gov/> (2012).

310 Vozzo, J. A. & Hacskaylo, E. Inoculation of Pinus caribaea with ectomycorrhizal fungi in Puerto Rico. *Forest Science* **17**, 239-245 (1971).

311 Rainer, G. *et al.* Host-Specialist Dominated Ectomycorrhizal Communities of Pinus cembra are not Affected by Temperature Manipulation. *Journal of Fungi* **1**, 55-75 (2015).

312 Byrd, K. B., Parker, V. T., Vogler, D. R. & Cullings, K. W. The influence of clear-cutting on ectomycorrhizal fungus diversity in a lodgepole pine (<i>Pinus contorta</i>) stand, Yellowstone National Park, Wyoming, and Gallatin National Forest, Montana. *Canadian Journal of Botany* **78**, 149-156 (2000).

313 Garcia, M. O., Smith, J. E., Luoma, D. L. & Jones, M. D. Ectomycorrhizal communities of ponderosa pine and lodgepole pine in the south-central Oregon pumice zone. *Mycorrhiza* **26**, 275-286 (2016).

314 Walker, C. Endogone-Lactiflua Forming Ectomycorrhizas with Pinus-Contorta. *T Brit Mycol Soc* **84**, 353-355 (1985).

315 Ma, D., Yang, G. & Mu, L. Morphological and molecular analyses of ectomycorrhizal diversity in Pinus densiflora seedlings. *Symbiosis* **51**, 233-238 (2010).

316 Wu, B., Nara, K. & Hogetsu, T. Competition between ectomycorrhizal fungi colonizing Pinus densiflora. *Mycorrhiza* **9**, 151-159 (1999).

317 Yamada, A., Ogura, T. & Ohmasa, M. Cultivation of mushrooms of edible ectomycorrhizal fungi associated with Pinus densiflora by in vitro mycorrhizal synthesis. II. Morphology of mycorrhizas in open-pot soil. *Mycorrhiza* **11**, 67-81 (2001).

318 Flores-Renteria, L., Lau, M. K., Lamit, L. J. & Gehring, C. An elusive ectomycorrhizal fungus reveals itself: a new species of Geopora (Pyronemataceae) associated with Pinus edulis. *Mycologia* **106**, 13-263- (2014).

319 Gehring, C. A., Theimer, T. C., Whitham, T. G. & Keim, P. Ectomycorrhizal fungal community structure of pinyon pines growing in two environmental extremes. *Ecology* **79**, 1562-1572 (1998).

320 Lamb, R. J. & Richards, B. N. Some mycorrhizal fungi of Pinus radiata and P. elliottii var. elliottii in Australia. *T Brit Mycol Soc* **54**, 371-378 (1970).

321 Rincón, A., Felipe, M. R. d. & Fernández-Pascual, M. Inoculation of Pinus halepensis Mill. with selected ectomycorrhizal fungi improves seedling establishment 2 years after planting in a degraded gypsum soil. *Mycorrhiza* **18**, 23-32 (2007).

322 Rusca, T. A., Kennedy, P. G. & Bruns, T. D. The effect of different pine hosts on the sampling of Rhizopogon spore banks in five Eastern Sierra Nevada forests. *New Phytol* **170**, 551-560 (2006).

323 Ajungla, T. & Sharma, G. D. Neutralization of heavy metals toxicity by Cenococcum graniforme in the establishment and survival of Pinus kesiya (Royle ex Gordon) seedlings. *International Journal of life sciences Biotechnology and Pharma Research* **2**, 279-283 (2013).

324 Rao, C. S., Sharma, G. D. & Shukla, A. K. Distribution of ectomycorrhizal fungi in pure stands of different age groups of Pinus kesiya. *Canadian Journal of Microbiology* **43**, 85-91 (1997).

325 Malysheva, E. F. *et al.* Below-Ground Ectomycorrhizal Community Structure in the Postfire Successional Pinus koraiensis Forests in the Central Sikhote-Alin (the Russian Far East). *Botanica Pacifica* **5**, 1-13 (2016).

326 Plamboeck, A. H. *et al.* Water transfer via ectomycorrhizal fungal hyphae to conifer seedlings. *Mycorrhiza* **17**, 439-447 (2007).

327 Bidartondo, M. I., Baar, J. & Bruns, T. D. Low ectomycorrhizal inoculum potential and diversity from soils in and near ancient forests of bristlecone pine (Pinus longaeva). *Canadian Journal of Botany* **79**, 293-299 (2001).

328 Huang, J. *et al.* Ectomycorrhizal fungal communities associated with Masson pine (Pinus massoniana Lamb.) in Pb-Zn mine sites of central south China. *Mycorrhiza* **22**, 589-602 (2012).

329 Reverchon, F., del Pilar Ortega-Larrocea, M., Bonilla-Rosso, G. & Pérez-Moreno, J. Structure and species composition of ectomycorrhizal fungal communities colonizing seedlings and adult trees of Pinus montezumae in Mexican neotropical forests. *FEMS Microbiology Ecology* **80**, 479-487 (2012).

330 Aučina, A. *et al.* Ectomycorrhizal fungal communities on seedlings and conspecific trees of Pinus mugo grown on the coastal dunes of the Curonian Spit in Lithuania. *Mycorrhiza* **21**, 237-245 (2011).

331 Horton, T. R., Cázares, E. & Bruns, T. D. Ectomycorrhizal, vesicular-arbuscular and dark septate fungal colonization of bishop pine (Pinus muricata) seedlings in the first 5 months of growth after wildfire. *Mycorrhiza* **8**, 11-18 (1998).

332 Horton, T. R. & Bruns, T. D. Multiple-host fungi are the most frequent and abundant ectomycorrhizal types in a mixed stand of Douglas fir (Pseudotsuga menziesii) and bishop pine (Pinus muricata). *New Phytol* **139**, 331-339 (1998).

333 Hawley, G. L., Taylor, A. F. S. & Dames, J. F. Ectomycorrhizas in association with Pinus patula in Sabie, South Africa. *South African Journal of Science* **104**, 273-283 (2008).

334 Dames, J. F., Straker, C. J. & Scholes, M. C. Ecological and anatomical characterization of some Pinus patula ectomycorrhizas from Mpumalanga, South Africa. *Mycorrhiza* **9**, 9-24 (1999).

335 Pera, J. & Alvarez, I. F. Ectomycorrhizal fungi of Pinus pinaster. *Mycorrhiza* **5**, 193-200 (1995).

336 Rincón, A., Álvarez, I. F. & Pera, J. Ectomycorrhizal fungi of Pinus pinea L. in northeastern Spain. *Mycorrhiza* **8**, 271-276 (1999).

337 Fujimura, K. E., Smith, J. E., Horton, T. R., Weber, N. S. & Spatafora, J. W. Pezizalean mycorrhizas and sporocarps in ponderosa pine (Pinus ponderosa) after prescribed fires in eastern Oregon, USA. *Mycorrhiza* **15**, 79-86 (2005).

338 Koizumi, T. & Nara, K. Two new species of Rhizopogon associated with Pinus pumila from Japan. *Mycoscience* (2016).

339 Verma, B. & Sudhakara Reddy, M. Suillus triacicularis sp. nov., a new species associated with Pinus roxburghii from northwestern Himalayas, India. *Phytotaxa* **162**, 157-164 (2014).

340 Smith, M. E., Douhan, G. W., Fremier, A. K. & Rizzo, D. M. Are true multihost fungi the exception or the rule? Dominant ectomycorrhizal fungi on Pinus sabiniana differ from those on co-occurring Quercus species. *New Phytol* **182**, 295-299 (2009).

341 Cowden, C. C. & Peterson, C. J. Annual and seasonal dynamics of ectomycorrhizal fungi colonizing white pine (Pinus strobus) seedlings following catastrophic windthrow in northern Georgia, USA. *Canadian Journal of Forest Research* **43**, 215-223 (2013).

342 Burke, D. J., Martin, K. J., Rygiewicz, P. T. & Topa, M. A. Relative abundance of ectomycorrhizas in a managed loblolly pine (Pinus taeda) genetics plantation as determined through terminal restriction fragment length polymorphism profiles. *Canadian Journal of Botany* **84**, 924-932 (2006).

343 Lewis, J. D. & Strain, B. R. The role of mycorrhizas in the response of Pinus taeda seedhngs to elevated CO2. *New Phytol* **133**, 431-443 (1996).

344 Matsuda, Y., Noguchi, Y. & Ito, S. i. Ectomycorrhizal fungal community of naturally regenerated Pinus thunbergii seedlings in a coastal pine forest. *Journal of Forest Research* **14**, 335-341 (2009).

345 Panaccione, D. G., Sheets, N. L., Miller, S. P. & Cumming, J. R. Diversity of Cenococcum geophilum isolates from serpentine and non-serpentine soils. *Mycologia* **93**, 645-652 (2001).

346 Hacskaylo, E. Thelephora terrestris and mycorrhizae of Virginia pine. *Forest Science* **11**, 401-404 (1965).

347 Tyub, S. et al. ITS-based molecular profiling of root-tips identifies novel ectomycorrhizae associated with Pinus wallichiana A.B. Jackson in temperate forests of Kashmir Himalaya (Genbank accession numbers: KP109910-KP109910), <http://www.ncbi.nlm.nih.gov/> (2014).

348 Hassan, D. G., A, B. M., A, G. N. & A, Q. N. Effect of source and inoculum load of ectomycorrhizae on the growth and biomass of containerized kail pine (Pinus wallichiana) seedlings. *Applied Biological Research* **9**, 19-28 (2007).

349 O'Brien, M. J., Gomola, C. E. & Horton, T. R. The effect of forest soil and community composition on ectomycorrhizal colonization and seedling growth. *Plant Soil* **341**, 321-331 (2011).

350 Lorillou, S. S. & Martin, F. F. *Cloning, sequence analysis and expression of a cDNA encoding NADP-glutamate dehydrogenase from the ectomycorrhizal basidiomycete Laccaria bicolor (Genbank accession: U31369)*, <www.ncbi.nlm.nih.gov> (1995).

351 Rimington, W. R., Pressel, S., Duckett, J. G. & Bidartondo, M. I. Fungal associations of basal vascular plants: reopening a closed book? *New Phytol* **205**, 1394-1398 (2015).

352 Strullu-Derrien, C. *et al.* Fungal associations in Horneophyton ligneri from the Rhynie Chert (c. 407 million year old) closely resemble those in extant lower land plants: Novel insights into ancestral plant-fungus symbioses. *New Phytol* **203**, 964-979 (2014).

353 Troia, A. & Greuter, W. A critical conspectus of Italian Isoetes (Isoetaceae). *Plant Biosystems* **148**, 13-20 (2014).

354 Taylor, L. L. *et al.* Biological weathering and the long-term carbon cycle: Integrating mycorrhizal evolution and function into the current paradigm. *Geobiology* **7**, 171-191 (2009).

355 Martínez-García, L. B., Richardson, S. J., Tylianakis, J. M., Peltzer, D. A. & Dickie, I. A. Host identity is a dominant driver of mycorrhizal fungal community composition during ecosystem development. *New Phytol* **205**, 1565-1576 (2015).

356 Ogura-Tsujita, Y. *et al.* Arbuscular mycorrhizal colonization in field-collected terrestrial cordate gametophytes of pre-polypod leptosporangiate ferns (Osmundaceae, Gleicheniaceae, Plagiogyriaceae, Cyatheaceae). *Mycorrhiza* **26**, 87-97 (2016).

357 Kai, W. & Zhiwei, Z. Occurrence of arbuscular mycorrhizas and dark septate endophytes in hydrophytes from lakes and streams in southwest China. *International Review of Hydrobiology* **91**, 29-37 (2006).

Table S3 | Taxon sampling and Genbank accession numbers of the data used for the phylogenetic inferrence of Embryophytes.

		Genbank accession		
**species (phylogenetic reconstruction)**	**Used in ancestral state reconstruction**	**rbcL**	**psbA**	**rps4**
Abies_alba	+	AB029652	NA	NA
Abies_balsamea	+	JN935605	NA	NA
Abies_homolepis	+	AB015648	NA	AY188224
Abies_magnifica	+	X58391	NA	NA
Abies_nordmanniana	+	AB029654	NA	NA
Abies_religiosa	+	JN935629	NA	NA
Acacia_mangium	+	KJ440053	NA	NA
Acanthus_ilicifolius	+	KF848219	NA	NA
Acer_macrophyllum	+	DQ978414	NA	NA
Acer_platanoides	+	DQ978424	NA	NA
Acer_pseudoplatanus	+	DQ978425	NA	NA
Achillea_millefolium	+	EU384938	NA	NA
Acianthus_exsertus	+	AF074101	NA	NA
Adiantum_capillus_veneris	-	NC_004766	NC_004766	NC_004766
Aegiceras_corniculatum	+	AY289687	NA	NA
Aegopodium_podagraria	+	U50220	NA	NA
Afrocarpus_falcatus	+	X58135	NA	AY188254
Afzelia_africana	+	KJ735963	NA	NA
Agarista_salicifolia	+	AF124588	NA	NA
Agrostis_capillaris	+	AY395527	NA	NA
Agrostis_scabra	+	JX848486	NA	NA
Agrostis_stolonifera	+	NC_008591	NC_008591	NC_008591
Ajuga_reptans	+	NC_023102	NC_023102	NC_023102
Allisonia_cockaynii	+	AY507389	AY507472	AY507432
Allium_ampeloprasum	+	HM849754	NA	NA
Allium_cepa	+	NC_024813	NC_024813	NC_024813
Allium_fistulosum	+	JQ273895	JQ275949	JQ274288
Alnus_acuminata	+	KF418925	NA	NA
Alnus_alnobetula	+	AY263926	NA	EU002289
Alnus_cordata	+	FJ844569	NA	NA
Alnus_glutinosa	+	KM360625	NA	NA
Alnus_incana	+	HM849757	NA	NA
Alnus_nitida	+	KF418931	NA	NA
Alnus_rubra	+	KF418933	NA	NA
Alsophila_spinulosa	-	NC_012818	NC_012818	NC_012818
Alzatea_verticillata	+	U26316	NA	NA
Amborella_trichopoda	-	NC_005086	NC_005086	NC_005086
Ammophila_arenaria	+	JN890705	NA	NA
Ammophila_breviligulata	+	AJ784835	NA	NA
Anacamptis_laxiflora	+	KF997401	NA	NA
Anacamptis_morio	+	KF997322	NA	NA
Anagallis_arvensis	+	M88343	NA	NA
Andromeda_polifolia	+	AF124572	NA	NA
Andropogon_gerardii	+	AJ784818	NA	NA
Anemone_patens	+	KR297057	KR297057	KR297057
Aneura_maxima	+	EF547813	NA	DQ983855
Aneura_mirabilis	+	NC_010359	NC_010359	NC_010359
Aneura_pinguis	+	AB476550	AY507474	AY608043
Angiopteris_angustifolia	-	NC_026300	NC_026300	NC_026300
Angiopteris_evecta	-	NC_008829	NC_008829	NC_008829
Anisoptera_costata	+	KJ594581	NA	NA
Anogramma_leptophylla	+	AY168715	NA	NA
Antennaria_dioica	+	KF602143	NA	NA
Anthoceros_agrestis	+	DQ845682	NA	KP238684
Anthoceros_angustus	-	NC_004543	NC_004543	NC_004543
Anthoceros_fusiformis	+	DQ845677	NA	NA
Anthoceros_lamellatus	+	DQ845679	NA	KP238690
Anthoceros_laminiferus	+	AY463053	NA	KP238691
Anthoceros_punctatus	+	U87063	NA	AJ250117
Anthoxanthum_odoratum	+	AJ746256	NA	Z29228
Aphyllorchis_montana	+	FJ454874	NA	NA
Arachis_hypogaea	+	NC_026676	NC_026676	NC_026676
Araucaria_angustifolia	+	U87750	NA	EU165034
Arbutus_unedo	+	JQ067650	JQ067650	JQ067650
Arctostaphylos_uva-ursi	+	GU176649	NA	NA
Aristida_adscensionis	+	EF423002	NA	NA
Arnica_montana	+	KF602249	NA	NA
Arrhenatherum_elatius	+	AJ784823	NA	NA
Artemisia_frigida	+	NC_020607	NC_020607	NC_020607
Artemisia_ludoviciana	+	JX848405	NA	NA

Artemisia_vulgaris	+	KM360653	NA	NA
Arthrocnemum_macrostachyum	+	AY270058	NA	NA
Arum_italicum	+	GU067578	NA	X84113
Arum_maculatum	+	GU067582	NA	NA
Arundina_graminifolia	+	AF074111	NA	NA
Asparagus_officinalis	+	HQ182417	HQ181820	JQ274297
Aster_tripolium	+	JN891980	NA	NA
Athyrium_yokoscense	+	D43893	NA	JN168078
Austrobaileya_scandens	+	L12632	NA	AF313613
Austrocedrus_chilensis	+	EU161449	NA	EU161501
Austrofossombronia_australis	+	AY507392	AY507475	HM036244
Avena_barbata	+	HM849803	NA	NA
Avicennia_officinalis	+	KF848224	NA	NA
Barbilophozia_barbata	+	DQ312477	AM396187	AM398313
Barbilophozia_hatcheri	+	DQ312478	KC184777	AM398338
Barbilophozia_lycopodioides	+	KC297121	KC184778	AM398333
Betula_alleghaniensis	+	KF418936	NA	NA
Betula_papyrifera	+	EU676902	NA	NA
Betula_pendula	+	KM360670	NA	NA
Betula_platyphylla	+	AY263927	NA	NA
Betula_pubescens	+	JN893073	NA	NA
Bistorta_vivipara	+	FM883608	NA	NA
Blasia_pusilla	+	AB476555	AY507477	NA
Boscia_foetida	+	JF265309	NA	NA
Botrychium_virginianum	+	AY138407	NA	NA
Bouteloua_gracilis	+	JX848489	NA	NA
Brachypodium_pinnatum	+	AY632361	NA	Z29230
Brassica_napus	+	NC_016734	NC_016734	NC_016734
Briza_media	+	KF602072	NA	NA
Brodiaea_coronaria	+	Z69210	NA	NA
Bromus_erectus	+	AJ746286	NA	Z29232
Bromus_tectorum	+	KF712969	AY744775	NA
Bucegia_romanica	+	EU519194	NA	AJ250182
Burmannia_capitata	+	AY149347	NA	NA
Cajanus_cajan	+	EU717273	NA	NA
Calamagrostis_arundinacea	+	HQ600475	NA	NA
Callicarpa_americana	+	KJ773329	NA	NA
Calluna_vulgaris	+	L12601	NA	NA
Caltha_palustris	+	EU053906	NA	NA
Calypogeia_fissa	+	DQ312479	NA	NA
Calypogeia_muelleriana	+	AM392302	AM396306	AY608052
Camellia_japonica	+	AF380035	NA	NA
Campanula_punctata	+	EU643725	NA	NA
Caragana_korshinskii	+	FJ537215	NA	NA
Cardamine_pratensis	+	KM360692	NA	NA
Carduus_tenuiflorus	+	HM849849	NA	NA
Carex_hirta	-	KJ841178	NA	NA
Carya_illinoinensis	+	KP088511	NA	NA
Castanea_dentata	+	KF418892	NA	NA
Castanea_mollissima	+	NC_014677	NC_014676	NC_014678
Castanea_sativa	+	KM360699	NA	NA
Castanopsis_fargesii	+	JF941175	NA	NA
Casuarina_equisetifolia	+	AY263930	NA	NA
Cecropia_obtusa	+	JQ626251	NA	NA
Cedrela_odorata	+	AJ402938	NA	NA
Cedrus_atlantica	+	AF145457	NA	EF053196
Cedrus_deodara	+	NC_014575	NC_014575	NC_014575
Cenchrus_ciliaris	+	FR821344	NA	NA
Centaurea_jacea	+	KF602073	NA	NA
Cephalanthera_damasonium	+	AF074123	NA	NA
Cephalanthera_erecta	+	JF972908	NA	NA
Cephalanthera_falcata	+	JF972909	NA	NA
Cephalanthera_longibracteata	+	AB856507	NA	NA
Cephalanthera_longifolia	+	JX051381	NA	NA
Cephalanthera_rubra	+	JX088502	NA	NA
Cephalozia_bicuspidata	+	KF852370	AM396186	JF513486
Cephaloziella_varians	+	DQ439689	AY607953	AY608074
Ceratopteris_richardii	-	KM052729	KM052729	KM052729
Cercocarpus_ledifolius	+	U06796	NA	NA
Chamaecyparis_obtusa	+	JQ512525	NA	AY188283
Chamerion_angustifolium	+	KM360765	NA	NA
Chimaphila_umbellata	+	AF419818	NA	NA
Cinchona_officinalis	+	AY538480	NA	NA
Cinnamomum_bejolghota	+	GQ248569	NA	NA
Citrullus_colocynthis	+	HM849900	NA	NA
Citrus_maxima	+	AB505955	NA	NA
Clematis_vitalba	+	KM360724	NA	NA

Clintonia_borealis	+	AB056856	NA	NA
Cneorum_tricoccon	+	FR747837	NA	NA
Coffea_arabica	+	NC_008535	NC_008535	NC_008535
Comarostaphylis_arbutoides	+	AF419819	NA	NA
Commelina_benghalensis	+	L05033	NA	NA
Conocephalum_conicum	+	AY688778	AB020574	DQ220678
Convallaria_majalis	+	D28334	NA	NA
Conyza_canadensis	+	KM360730	NA	NA
Corallorhiza_trifida	+	NC_025662	NC_025662	NC_025662
Cornus_suecica	+	AF421085	JF321223	NA
Corycium_carnosum	+	AY381115	NA	NA
Corylus_avellana	+	HM849918	NA	NA
Corylus_colurna	+	KF418951	NA	NA
Crataegus_monogyna	+	KM360737	NA	NA
Cremastra_appendiculata	+	FJ445514	NA	NA
Cymbidium_ensifolium	+	AF074141	NA	NA
Cymbidium_floribundum	+	KJ597961	NA	NA
Cymbidium_goeringii	+	KC704878	NA	NA
Cymbidium_lancifolium	+	KJ597953	NA	NA
Cymbidium_macrorhizon	+	KF296680	NA	NA
Cymbidium_sinense	+	NC_021430	NC_021430	NC_021430
Cymbopogon_nardus	+	KJ740992	NA	NA
Cynara_cardunculus	+	KM035764	KM035764	KM035764
Cynosurus_cristatus	+	HM849932	NA	NA
Cynosurus_echinatus	+	HM849933	NA	NA
Cypripedium_acaule	+	EF370111	NA	NA
Cypripedium_arietinum	+	EF370104	NA	NA
Cypripedium_calceolus	+	AB176549	NA	NA
Cypripedium_californicum	+	EF370108	NA	NA
Cypripedium_candidum	+	EF370103	NA	NA
Cypripedium_debile	+	EF370114	NA	NA
Cypripedium_fasciculatum	+	EF370112	NA	NA
Cypripedium_flavum	+	JN181474	NA	NA
Cypripedium_formosanum	+	NC_026772	NC_026772	NC_026772
Cypripedium_guttatum	+	EF370113	NA	NA
Cypripedium_japonicum	+	KJ625630	KJ625630	KJ625630
Cypripedium_montanum	+	EF370102	NA	NA
Cypripedium_parviflorum	+	EF370106	NA	NA
Cypripedium_reginae	+	EF370105	NA	NA
Cypripedium_tibeticum	+	JQ182215	NA	NA
Cystopteris_protrusa	-	KP136830	KP136830	KP136830
Dacrycarpus_dacrydioides	+	AF249597	NA	NA
Dactylis_glomerata	+	JX848494	NA	NA
Daucus_carota	+	NC_008325	NC_008325	NC_008325
Delonix_regia	+	AY904419	NA	NA
Dendrobium_catenatum	+	KF177581	HM768333	NA
Dendrobium_chrysanthum	+	KF177586	NA	NA
Dendrobium_crumenatum	+	JF713166	NA	NA
Dendrobium_fimbriatum	+	KF177603	NA	NA
Dendrobium_nobile	+	AB519785	NA	NA
Dendroceros_crispus	+	DQ845662	NA	KP238702
Dendroceros_validus	+	JX885634	NA	NA
Dendrolycopodium_dendroideum	+	KJ593352	NA	NA
Dichrostachys_cinerea	+	JQ025041	NA	NA
Dicksonia_squarrosa	+	KJ569698	KJ569698	KJ569698
Dicorynia_guianensis	+	JQ626129	NA	NA
Dimocarpus_longan	+	AY724349	NA	NA
Dioon_edule	-	AF531203	NA	GQ204075
Dioscorea_rotundata	+	KJ629262	JF705476	NA
Diphasiastrum_alpinum	+	AJ133250	NA	AM777778
Diplophyllum_albicans	+	AM392309	AM396190	AY608060
Diplophyllum_obtusifolium	+	AY507397	AY507480	AY507439
Diplopterygium_glaucum	+	NC_024158	NC_024158	NC_024158
Dipteris_conjugata	-	KP136829	KP136829	KP136829
Dipterocarpus_tuberculatus	+	KM267148	NA	NA
Disa_bracteata	+	AY368344	NA	NA
Disperis_capensis	+	AY381120	NA	NA
Dodonaea_viscosa	+	AM235129	NA	NA
Dryas_integrifolia	+	KC482649	NA	NA
Dryas_octopetala	+	KF602200	NA	JF317523
Dysphania_ambrosioides	+	HM587599	NA	NA
Elephantopus_scaber	+	GQ436445	NA	NA
Empetrum_nigrum	+	AF421091	NA	NA
Enkianthus_campanulatus	+	L12616	NA	NA
Enkianthus_cernuus	+	AB726263	NA	NA
Enkianthus_nudipes	+	AB726264	NA	NA
Enkianthus_perulatus	+	JF941463	NA	NA

Enkianthus_sikokianus	+	AB726257	NA	NA
Enkianthus_subsessilis	+	AB726255	NA	NA
Eperua_falcata	+	JQ626198	NA	NA
Eperua_grandiflora	+	JQ625939	NA	NA
Ephedra_fragilis	+	AY755784	NA	AY591477
Epidendrum_rhopalostele	+	KF679618	NA	NA
Epilobium_ciliatum	+	KF997326	NA	NA
Epipactis_albensis	+	FJ454878	NA	NA
Epipactis_atrorubens	+	JX088503	NA	NA
Epipactis_dunensis	+	JX088504	NA	NA
Epipactis_helleborine	+	Z73707	NA	NA
Epipactis_palustris	+	JX094817	NA	NA
Epipactis_purpurata	+	JX094816	NA	NA
Equisetum_arvense	-	NC_014699	NC_014699	NC_014699
Equisetum_fluviatile	+	AB574684	NA	DQ463116
Equisetum_hyemale	-	NC_020146	NC_020146	NC_020146
Erica_carnea	+	KP737713	NA	NA
Erica_ciliaris	+	KF997417	NA	NA
Erica_cinerea	+	KM360768	NA	NA
Erica_vagans	+	KF997402	NA	NA
Eriochilus_cucullatus	+	AF074166	NA	NA
Erythranthe_guttata	+	KF997284	NA	KJ161985
Eucalyptus_camaldulensis	+	NC_022398	NC_022398	NC_022398
Eucalyptus_globulus	+	NC_008115	NC_008115	NC_008115
Eucalyptus_gunnii	+	KM360776	NA	NA
Eucalyptus_marginata	+	NC_022390	NC_022390	NC_022390
Eucalyptus_nitens	+	NC_022395	NC_022395	NC_022395
Excoecaria_agallocha	+	AY794839	NA	NA
Exormotheca_pustulosa	+	DQ286010	DQ265754	DQ220684
Fagopyrum_esculentum	+	NC_010776	NC_010776	NC_010776
Fagopyrum_tataricum	+	NC_027161	NC_027161	NC_027161
Fagus_grandifolia	+	AY935745	NA	NA
Fagus_sylvatica	+	KF418911	NA	NA
Faramea_occidentalis	+	JQ593668	NA	NA
Festuca_idahoensis	+	KJ756344	NA	NA
Festuca_ovina	+	NC_019649	NC_019649	NC_019649
Festuca_pratensis	+	NC_019650	NC_019650	NC_019650
Festuca_rubra	+	AJ746261	NA	NA
Festuca_rupicola	+	KJ746319	NA	NA
Folioceros_fuciformis	+	AY463050	NA	KP238708
Folioceros_glandulosus	+	JF815573	NA	NA
Fossombronia_angulosa	+	AY507398	AY507481	HQ447031
Fragaria_vesca	+	NC_015206	NC_015206	NC_015206
Fraxinus_excelsior	+	KP088611	NA	NA
Galium_album	+	X81090	NA	NA
Galium_aparine	+	X81091	NA	HQ385112
Gaultheria_procumbens	+	HQ590116	NA	NA
Gaultheria_shallon	+	KM360799	NA	NA
Genista_cinerea	+	Z70094	NA	NA
Geothallus_tuberosus	+	U87070	NA	NA
Geranium_pratense	+	KP963381	KP963305	NA
Geum_rivale	+	KM360804	NA	NA
Ginkgo_biloba	+	NC_016986	NC_016986	NC_016986
Glechoma_hederacea	+	Z37391	NA	NA
Gleichenia_microphylla	+	DQ910504	NA	JQ911728
Gliricidia_sepium	+	KF381127	NA	NA
Glycine_max	+	NC_007942	NC_007942	NC_007942
Gnetum_gnemon	+	NC_026301	NC_026301	NC_026301
Goodyera_foliosa	+	HM141074	NA	NA
Goodyera_maximowicziana	+	KF296607	NA	NA
Goodyera_oblongifolia	+	HM141070	NA	NA
Goodyera_procera	+	HM141076	NA	NA
Goodyera_pubescens	+	FJ571329	NA	NA
Goodyera_repens	+	FJ571330	NA	NA
Goodyera_schlechtendaliana	+	HM141073	NA	NA
Goodyera_velutina	+	HM141075	NA	NA
Guapira_discolor	+	KJ522686	NA	NA
Guapira_fragrans	+	JX844257	NA	NA
Guarea_pterorhachis	+	JQ592716	NA	NA
Gymnadenia_conopsea	+	KC704906	NA	NA
Gynostemma_pentaphyllum	+	AY968523	NA	NA
Handroanthus_ochraceus	+	JQ590852	NA	NA
Haplomitrium_blumei	+	AY507402	AY507485	AY507443
Haplomitrium_chilensis	+	KC305698	NA	NA
Haplomitrium_gibbsiae	+	KF852350	AY607947	AY688793
Haplomitrium_hookeri	+	U87072	AY877398	AJ251064
Hedera_rhombea	+	AB817831	NA	NA

Hedyosmum_goudotianum	+	EU302198	NA	NA
Helianthemum_squamatum	+	FJ225851	NA	NA
Helianthus_annuus	+	NC_007977	NC_007977	NC_007977
Helichrysum_stoechas	+	GU817766	NA	NA
Heteropogon_contortus	+	AM235061	NA	NA
Heterotheca_villosa	+	JX848417	NA	NA
Hexalectris_revoluta	+	AF264168	FJ457818	NA
Hieronyma_oblonga	+	AY663588	NA	NA
Holcus_lanatus	+	KF602083	NA	NA
Hopea_odorata	+	KM267144	NA	NA
Hordeum_vulgare	+	NC_008590	NC_008590	NC_008590
Huperzia_appressa	+	DQ464220	NA	NA
Huperzia_lucidula	+	NC_006861	NC_006861	NC_006861
Hyacinthoides_non-scripta	+	KM360826	NA	NA
Hymenostegia_ngouniensis	+	KC685085	NA	NA
Hypericum_maculatum	+	KF602180	NA	NA
Hypochaeris_radicata	+	AY395542	NA	NA
Inga_acreana	+	JQ625968	NA	NA
Inga_edulis	+	FJ173737	NA	NA
Intsia_bijuga	+	KF496786	NA	NA
Inula_conyza	+	JN892414	NA	NA
Ionopsis_utricularioides	+	FJ534225	NA	NA
Ipomoea_pes_caprae	+	KF242490	KF242490	KF242490
Isoetes_echinospora	+	FJ785184	NA	NA
Isoetes_flaccida	-	NC_014675	NC_014675	NC_014675
Isoetes_lacustris	+	AJ010855	NA	NA
Isopaches_bicrenatus	+	KF852384	KF851946	KF851475
Jacobaea_vulgaris	+	NC_015543	NC_015543	NC_015543
Japonolirion_osense	+	JQ068978	NA	NA
Jensenia_connivens	+	AY688782	AY688829	AY507450
Juglans_regia	+	KF418920	NA	NA
Juncus_bulbosus	+	HM850086	NA	NA
Kandelia_candel	+	AF127682	NA	NA
Keteleeria_davidiana	+	NC_011930	NC_011930	NC_011930
Knautia_arvensis	+	KF602109	NA	NA
Kobresia_myosuroides	+	KF602151	NA	NA
Kummerowia_striata	+	KJ773609	NA	NA
Larix_decidua	+	NC_016058	NC_016058	NC_016058
Larix_gmelinii	+	JQ512557	NA	EF053200
Larix_kaempferi	+	JQ512558	NA	NA
Lavandula_latifolia	+	Z37406	NA	NA
Leiosporoceros_dussii	+	AY463052	NA	KP238712
Leontodon_hispidus	+	AY395545	NA	NA
Lepidozia_reptans	+	AM392313	AM396195	AY608083
Leymus_mollis	+	KF712997	NA	NA
Ligularia_virgaurea	+	JF942280	NA	NA
Ligustrum_vulgare	+	DQ673302	NA	HQ385090
Limodorum_abortivum	+	JX051376	NA	NA
Linnaea_borealis	+	KM360854	KP297623	GQ983907
Liparis_kumokiri	+	KC704909	NA	NA
Liparis_liliifolia	+	AF074183	NA	NA
Liparis_loeselii	+	KM360856	NA	NA
Listera_cordata	+	JN965648	NA	NA
Listera_ovata	+	JX051379	NA	NA
Litsea_cubeba	+	AY337734	NA	NA
Littorella_uniflora	+	HM850128	NA	NA
Lobatiriccardia_lobata	+	AY507421	AY507507	AY507462
Lobelia_dortmanna	+	DQ356162	NA	NA
Lolium_multiflorum	+	NC_019651	NC_019651	NC_019651
Lolium_perenne	+	NC_009950	NC_009950	NC_009950
Lophozia_ventricosa	+	AY699994	KF851831	JF513477
Lophozonia_cunninghamii	+	AY605490	NA	NA
Lophozonia_menziesii	+	AY605494	NA	NA
Lotus_brunneri	+	KM372996	NA	NA
Lotus_corniculatus	+	KM360864	NA	NA
Lotus_jacobaeus	+	KM373014	NA	NA
Lotus_japonicus	+	NC_002694	NC_002694	NC_002694
Luehea_seemannii	+	GQ981791	NA	NA
Lupinus_latifolius	+	Z70059	NA	NA
Luronium_natans	+	U80680	NA	NA
Lycium_barbarum	+	KP088673	NA	NA
Lycopodiella_inundata	+	AB574631	NA	NA
Lycopodiella_lateralis	+	AJ133262	NA	NA
Lycopodium_annotinum	+	AJ133247	NA	AM777801
Lycopodium_clavatum	+	Y07936	NA	DQ463115
Lycopodium_fastigiatum	+	AJ133252	NA	AM777805
Lycopodium_volubile	+	AJ133253	NA	AM777808

Lygodium_japonicum	-	NC_022136	NC_022136	NC_022136
Maianthemum_bifolium	+	EU850093	NA	NA
Maianthemum_racemosum	+	AY149376	NA	NA
Malus_hupehensis	+	JQ391361	NA	NA
Malus_micromalus	+	GU363816	NA	NA
Malus_sieboldii	+	JQ391372	NA	NA
Mangifera_indica	+	GQ436547	NA	NA
Mankyua_chejuensis	-	NC_017006	NC_017006	NC_017006
Mannia_androgyna	+	AM920276	DQ265756	DQ220686
Mannia_fragrans	+	DQ286013	DQ265757	DQ220687
Marchantia_paleacea	+	DQ286015	DQ265759	DQ220689
Marsilea_crenata	+	NC_022137	NC_022137	NC_022137
Medicago_murex	+	KC700638	NA	NA
Medicago_polymorpha	+	KJ773677	NA	NA
Medicago_truncatula	+	JX512022	JX512022	JX512022
Megaceros_flagellaris	+	GQ845371	AY877400	KP238713
Megaceros_leptohymenius	+	HM056150	NA	KP238714
Megaceros_pellucidus	+	GQ504733	NA	NA
Megathyrsus_maximus	+	AM849390	NA	NA
Melilotus_albus	+	DQ006095	NA	NA
Melinis_repens	+	EF125136	NA	Z29259
Mercurialis_perennis	+	KM360880	NA	NA
Metzgeria_conjugata	+	AY507411	AY507495	AY507453
Metzgeria_decipiens	+	AB535634	NA	AM398259
Metzgeria_furcata	+	AB535635	AM396271	DQ463119
Metzgeria_pubescens	+	AB535632	AM396272	DQ268982
Metzgeria_temperata	+	AB535636	NA	AB535657
Miscanthus_sinensis	+	EF125118	NA	NA
Monoclea_gottschei	+	DQ286016	DQ265760	AY507455
Monosolenium_tenerum	+	DQ286017	NA	DQ220691
Montinia_caryophyllacea	+	L11194	NA	HQ385127
Moringa_drouhardii	+	AF405249	NA	NA
Moringa_hildebrandtii	+	JX091930	NA	NA
Moringa_oleifera	+	JX091931	NA	NA
Musa_acuminata	+	HF677508	HF677508	HF677508
Myriopteris_lindheimeri	-	NC_014592	NC_014592	NC_014592
Nardia_compressa	+	DQ026586	NA	NA
Nardia_geoscyphus	+	KF943554	KF942493	KF943501
Nardia_scalaris	+	KF943606	AY607971	JF513481
Neohodgsonia_mirabilis	+	DQ286018	AY507499	DQ220692
Neoorthocaulis_floerkei	+	KC297118	KC184803	KF851449
Neottia_nidus-avis	+	AY368364	NA	NA
Nephrolepis_hirsutula	+	AB575229	NA	AY459163
Nervilia_nipponica	+	KF296711	NA	NA
Neuwiedia_veratrifolia	+	AF074200	NA	NA
Noccaea_caerulescens	+	JN891339	NA	NA
Nothoceros_aenigmaticus	-	NC_020259	NC_020259	NC_020259
Nothoceros_giganteus	+	AY463046	NA	KP238719
Nothoceros_vincentianus	+	AY463045	NA	KP238724
Notothylas_javanica	+	DQ845664	NA	KP238729
Notothylas_orbicularis	+	AY463055	NA	DQ463117
Nyholmiella_obtusifolia	+	NC_026979	NC_026979	NC_026979
Nymphaea_alba	+	NC_006050	NC_006050	NC_006050
Odontoschisma_denudatum	+	DQ645977	AY607975	KJ620750
Olea_europaea	+	NC_013707	NC_013707	NC_013707
Oloptum_miliaceum	+	FN870403	NA	NA
Ononis_repens	+	KF602196	NA	NA
Ophioglossum_californicum	-	NC_020147	NC_020147	NC_020147
Ophioglossum_costatum	+	AB626643	NA	NA
Ophioglossum_vulgatum	+	AB574681	JQ684697	NA
Ophrys_fuciflora	+	GQ248658	NA	NA
Ophrys_insectifera	+	HE858481	NA	NA
Orchis_anthropophora	+	KF997307	NA	NA
Orchis_mascula	+	KM360905	NA	NA
Orchis_militaris	+	KF997273	NA	NA
Orchis_purpurea	+	KF997502	NA	NA
Orchis_simia	+	KF997259	NA	NA
Origanum_vulgare	+	JX880022	JX880022	JX880022
Orixa_japonica	+	HE588085	NA	NA
Orthilia_secunda	+	AF419838	NA	NA
Orthocaulis_attenuatus	+	GU373417	NA	AM398282
Oryza_sativa	+	NC_001320	NC_001320	NC_001320
Osmunda_regalis	+	EF588705	NA	EF588768
Osmundastrum_cinnamomeum	-	NC_024157	NC_024157	NC_024157
Ostrya_carpinifolia	+	KF418956	NA	NA
Oxalis_acetosella	+	FJ670181	NA	NA
Oxalis_stricta	+	KC481639	NA	NA

Oxymitra_incrassata	+	EU519195	NA	EU519197
Pachyschistochila_splachnophylla	+	KF184409	NA	KF184437
Palhinhaea_cernua	+	KJ773661	NA	NA
Pallavicinia_xiphoides	+	AY734692	AY734709	AY734700
Panax_ginseng	+	NC_006290	NC_006290	NC_006290
Panax_japonicus	+	GQ436711	NA	NA
Panicum_virgatum	+	NC_015990	NC_015990	NC_015990
Paphiopedilum_armeniacum	+	NC_026779	NC_026779	NC_026779
Paphiopedilum_dianthum	+	JQ182210	NA	NA
Paraphymatoceros_coriaceus	+	AY463042	NA	NA
Paris_incompleta	+	JF942774	NA	NA
Paris_quadrifolia	+	KM360917	NA	NA
Paspalum_distichum	+	FN870399	NA	NA
Pellia_endiviifolia	+	NC_019628	NC_019628	NC_019628
Perilla_frutescens	+	FJ513160	NA	NA
Phaeoceros_carolinianus	+	DQ646009	NA	GQ428118
Phaeoceros_dendroceroides	+	KF482291	NA	KP238737
Phaeoceros_laevis	+	DQ845673	NA	KP238743
Phaeoceros_pearsonii	+	AY860203	NA	KP238734
Phaeomegaceros_coriaceus	+	JX872450	NA	KP238750
Phaeomegaceros_hirticalyx	+	KC958508	NA	KP238751
Phaius_tancarvilleae	+	KF852752	NA	NA
Phalaris_arundinacea	+	AJ784827	NA	NA
Phaseolus_vulgaris	+	NC_009259	NC_009259	NC_009259
Phellodendron_amurense	+	FN599455	NA	NA
Phlegmariurus_phlegmaria	+	AB574635	NA	NA
Phoenix_dactylifera	+	NC_013991	NC_013991	NC_013991
Phragmites_australis	+	NC_022958	NC_022958	NC_022958
Phyllanthus_calycinus	+	AY663603	NA	EU002353
Phyllothallia_nivicola	+	AY507418	AY507504	AY507459
Physcomitrella_patens	+	NC_005087	NC_005087	NC_005087
Phytolacca_americana	+	FJ860398	NA	HQ843413
Picea_abies	+	NC_021456	NC_021456	NC_021456
Picea_crassifolia	+	EF440576	NA	NA
Picea_glauca	+	JX508439	NA	NA
Picea_jezoensis	+	JQ512567	NA	NA
Picea_mariana	+	JX508447	NA	NA
Picea_rubens	+	EF440600	NA	NA
Picea_sitchensis	+	NC_011152	NC_011152	NC_011152
Picea_smithiana	+	AF145458	NA	AY188226
Pilea_pumila	+	AF206811	NA	NA
Pilosella_officinarum	+	KF602082	NA	NA
Pinus_albicaulis	+	FJ899566	FJ899566	FJ899566
Pinus_banksiana	+	FJ899571	FJ899571	FJ899571
Pinus_canariensis	+	FJ899572	FJ899572	FJ899572
Pinus_caribaea	+	JN854222	JN854222	JN854222
Pinus_cembra	+	FJ899574	NA	FJ899574
Pinus_contorta	+	NC_011153	NC_011153	NC_011153
Pinus_densiflora	+	JN854210	JN854210	JN854210
Pinus_edulis	+	JN854203	JN854203	JN854203
Pinus_elliottii	+	JN854202	JN854202	JN854202
Pinus_halepensis	+	JN854197	JN854197	JN854197
Pinus_jeffreyi	+	JN854193	JN854193	JN854193
Pinus_kesiya	+	JN854191	JN854191	JN854191
Pinus_koraiensis	+	NC_004677	NC_004677	NC_004677
Pinus_lambertiana	+	NC_011156	NC_011156	NC_011156
Pinus_longaeva	+	AF456383	NA	NA
Pinus_massoniana	+	NC_021439	NC_021439	NC_021439
Pinus_montezumae	+	JN854183	JN854183	JN854183
Pinus_mugo	+	JN854181	JN854181	JN854181
Pinus_muricata	+	JN854180	JN854180	JN854180
Pinus_nigra	+	JN854179	JN854179	JN854179
Pinus_parviflora	+	FJ899581	FJ899581	FJ899581
Pinus_patula	+	JN854175	JN854175	JN854175
Pinus_pinaster	+	FJ899583	FJ899583	FJ899583
Pinus_pinea	+	JN854173	JN854173	JN854173
Pinus_ponderosa	+	FJ899555	FJ899555	FJ899555
Pinus_pumila	+	JN854168	JN854168	JN854168
Pinus_radiata	+	JN854165	JN854165	JN854165
Pinus_roxburghii	+	JN854162	JN854162	JN854162
Pinus_sabiniana	+	JN854161	JN854161	JN854161
Pinus_strobus	+	NC_026302	NC_026302	NC_026302
Pinus_sylvestris	+	JN854158	JN854158	JN854158
Pinus_tabuliformis	+	AY555714	NA	NA
Pinus_taeda	+	NC_021440	NC_021440	NA
Pinus_thunbergii	+	NC_001631	NC_001631	NC_001631
Pinus_virginiana	+	JN854155	JN854155	JN854155

Pinus_wallichiana	+	JN854154	JN854154	JN854154
Piper_nigrum	+	AY298847	NA	DQ972915
Pisonia_aculeata	+	KJ594427	NA	NA
Pisonia_albida	+	JX844264	NA	NA
Pisonia_grandis	+	JX844258	NA	NA
Pisonia_sandwicensis	+	JX844266	NA	NA
Pisonia_sechellarum	+	JX844267	NA	NA
Pisonia_taina	+	JX844260	NA	NA
Pisum_sativum	+	NC_014057	NC_014057	NC_014057
Plagiogyria_glauca	-	KP136831	KP136831	KP136831
Plantago_afra	+	AJ389602	NA	NA
Plantago_asiatica	+	GQ436317	NA	NA
Plantago_lanceolata	+	HM850265	NA	NA
Plantago_major	+	HM850266	NA	NA
Platanthera_azorica	+	HM850267	NA	NA
Platanthera_chlorantha	+	JN891411	NA	NA
Platanthera_micrantha	+	HM850268	NA	NA
Pleurozia_gigantea	+	KF852349	AM396268	KF851436
Pleurozia_purpurea	+	AY877391	AY877401	AY608100
Poa_pratensis	+	JX848506	NA	NA
Podocarpus_oleifolius	+	HM593664	NA	NA
Podococcus_barteri	+	NC_027276	NC_027276	NC_027276
Podomitrium_phyllanthus	+	AY507419	AY507505	AY507460
Polygala_amara	+	Z70175	NA	NA
Polygala_calcarea	+	AM234194	NA	NA
Polygala_comosa	+	AM234211	NA	NA
Polygala_myrtifolia	+	AJ829699	NA	NA
Polygala_rupestris	+	AM234220	NA	NA
Polygala_serpyllifolia	+	EU644685	NA	NA
Polygala_vulgaris	+	AM234193	NA	NA
Polygonum_cuspidatum	+	AB019030	NA	NA
Polypodium_glycyrrhiza	-	KP136832	KP136832	KP136832
Polystichum_acrostichoides	+	AF537235	NA	NA
Populus_alba	+	NC_008235	NC_008235	NC_008235
Populus_balsamifera	+	NC_024735	NC_024735	NC_024735
Populus_davidiana	+	KC485208	NA	NA
Populus_deltoides	+	AJ418829	X78204	NA
Populus_euphratica	+	NC_024747	NC_024747	NC_024747
Populus_nigra	+	AJ418828	NA	NA
Populus_simonii	+	KF940819	NA	NA
Populus_tremula	+	NC_027425	NC_027425	NC_027425
Populus_trichocarpa	+	NC_009143	NC_009143	NC_009143
Potentilla_erecta	+	HM850285	NA	NA
Preissia_quadrata	+	AY312935	AY312916	AY608102
Prumnopitys_ferruginea	+	AF249656	NA	NA
Prumnopitys_montana	+	KF714220	NA	NA
Prumnopitys_taxifolia	+	HM593621	NA	AY188259
Prunella_vulgaris	+	AY395556	NA	NA
Prunus_africana	+	AM235108	NA	NA
Prunus_persica	+	NC_014697	NC_014697	NC_014697
Pseudorchis_albida	+	KF997412	NA	NA
Pseudotsuga_menziesii	+	JN854170	JN854170	JN854170
Psilotum_nudum	+	NC_003386	NC_003386	NC_003386
Pteridium_aquilinum	-	NC_014348	NC_014348	NC_014348
Pterostylis_nutans	+	AF074224	NA	NA
Pterygodium_catholicum	+	AY368346	NA	NA
Ptisana_purpurascens	+	EU439089	NA	EU439132
Puccinellia_distans	+	HE577877	NA	NA
Puccinellia_maritima	+	JN892490	NA	NA
Pulmonaria_obscura	+	EU599876	NA	NA
Purdiaea_nutans	+	AY082698	NA	NA
Pyrola_minor	+	KM360950	NA	NA
Pyrola_rotundifolia	+	JN892808	NA	NA
Pyrorchis_nigricans	+	AF074187	NA	NA
Pyrus_pyrifolia	+	NC_015996	NC_015996	NC_015996
Quercus_agrifolia	+	KF683137	NA	NA
Quercus_alba	+	KF418899	NA	NA
Quercus_cerris	+	FN675722	NA	NA
Quercus_douglasii	+	KF683145	NA	NA
Quercus_fabri	+	KP088808	NA	NA
Quercus_ilex	+	FN675717	NA	NA
Quercus_incana	+	KJ773803	NA	NA
Quercus_petraea	+	KF683156	NA	NA
Quercus_phellos	+	KJ773810	NA	NA
Quercus_pubescens	+	FN675720	NA	NA
Quercus_robur	+	KP088814	NA	NA
Quercus_rubra	+	NC_020152	NC_020152	NC_020152

Quercus_salicina	+	AB060570	NA	NA
Quercus_suber	+	FN675726	NA	NA
Quercus_variabilis	+	AB060574	NA	NA
Quercus_wislizeni	+	KF683168	NA	NA
Ranunculus_montanus	+	KF602172	NA	NA
Ranunculus_repens	+	HM850298	NA	NA
Retama_sphaerocarpa	+	Z70119	NA	NA
Rhizophora_apiculata	+	AF127685	NA	NA
Rhododendron_decorum	+	HQ706897	NA	NA
Rhododendron_ferrugineum	+	KF602219	NA	NA
Rhododendron_fortunei	+	HQ706905	NA	NA
Rhododendron_groenlandicum	+	AF419831	NA	NA
Rhododendron_racemosum	+	JF944013	NA	NA
Rhododendron_tomentosum	+	AF421101	NA	NA
Rhomboda_cristata	+	HM141068	NA	NA
Riccardia_latifrons	+	KC305702	NA	NA
Riccardia_multifida	+	DQ268980	NA	DQ268992
Riccardia_palmata	+	KC305704	NA	DQ986149
Riccia_huebeneriana	+	AY507422	AY507508	AY507463
Riella_helicophylla	+	DQ268981	NA	NA
Robinia_pseudoacacia	+	NC_026684	NC_026684	NC_026684
Rosa_multiflora	+	KP088837	NA	NA
Rosmarinus_officinalis	+	NC_027259	NC_027259	NC_027259
Rubus_parvifolius	+	GU363802	NA	NA
Rubus_saxatilis	+	JN891152	NA	NA
Ruta_chalepensis	+	HM850326	NA	NA
Saccobasis_polita	+	KF852308	KF851862	KF851413
Saccogyna_viticulosa	+	KF852385	AM396175	KP671947
Salicornia_europaea	+	NC_027225	NC_027225	NC_027225
Salix_alba	+	AB012780	NA	NA
Salix_arctica	+	KM002834	NA	NA
Salix_herbacea	+	KF602181	NA	NA
Salix_polaris	+	FJ788569	NA	NA
Salix_reinii	+	AB012792	NA	NA
Salix_reticulata	+	AJ235793	NA	NA
Salix_tetrasperma	+	AB012798	NA	NA
Salvadora_persica	+	X69755	NA	NA
Sanionia_uncinata	+	NC_025668	NC_025668	NC_025668
Santiria_trimera	+	GU246043	NA	NA
Saussurea_involucrata	+	GQ436481	NA	NA
Sauteria_alpina	+	DQ286025	DQ265768	HQ225532
Saxifraga_oppositifolia	+	JN965986	NA	NA
Scapania_calcicola	+	KC184746	KC184814	NA
Scapania_cuspiduligera	+	KC184748	KC184816	NA
Scapania_irrigua	+	KC305708	NA	NA
Scapania_umbrosa	+	KC184757	KC184825	NA
Schistochilopsis_incisa	+	AM392308	AM396188	AM398234
Schizocodon_soldanelloides	+	AF421105	NA	NA
Selaginella_kraussiana	+	AJ010845	NA	NA
Selaginella_moellendorffii	-	NC_013086	NC_013086	NC_013086
Selaginella_selaginoides	+	AF419048	NA	NA
Selaginella_uncinata	-	AB197035	AB197035	AB197035
Senegalia_senegal	+	JF265258	NA	NA
Sequoiadendron_giganteum	+	AY056580	NA	AY188267
Serapias_cordigera	+	HM850348	NA	NA
Sesleria_caerulea	+	EF125156	NA	X84142
Setaria_pumila	+	KF163544	NA	NA
Shorea_guiso	+	AB925523	NA	NA
Shorea_obtusa	+	AB925320	NA	NA
Solanum_tuberosum	+	NC_008096	NC_008096	NC_008096
Solidago_canadensis	+	KM360988	NA	NA
Solidago_gigantea	+	HM850369	NA	NA
Solidago_missouriensis	+	JX848425	NA	NA
Solidago_rugosa	+	EU677029	NA	NA
Solidago_virgaurea	+	KF602089	NA	NA
Sonchus_tenerrimus	+	HM850374	NA	NA
Sonneratia_alba	+	KF848248	NA	NA
Sorbus_aucuparia	+	HQ590284	NA	NA
Sorghum_bicolor	+	NC_008602	NC_008602	NC_008602
Southbya_nigrella	+	KF852355	KF851914	KF851446
Sphaerocarpos_texanus	+	AY507425	AY507511	NA
Sphagnum_palustre	+	AF231887	AY312920	JX241483
Spiranthes_sinensis	+	KC704924	NA	NA
Subularia_aquatica	+	KM361000	NA	NA
Swietenia_macrophylla	+	U39080	NA	EU002368
Symphyogyna_hymenophyllum	+	AY507426	AY507512	AY507467
Syntrichia_ruralis	+	NC_012052	NC_012052	NC_012052

Tacca_plantaginea	+	JQ733814	NA	NA
Tainia_latifolia	+	KF852754	NA	NA
Takakia_lepidozioides	+	AB299142	AY312921	AB299142
Tanacetum_vulgare	+	KM361004	NA	NA
Taraxacum_officinale	+	AY395562	NA	NA
Taxus_baccata	+	AF456388	NA	X84145
Tectona_grandis	+	NC_020098	NC_020098	NC_020098
Tetragastris_panamensis	+	JQ626012	NA	NA
Tetraphis_pellucida	+	NC_024291	NC_024291	NC_024291
Thalictrum_minus	+	EU053923	NA	KC289069
Thismia_rodwayi	+	AY939892	NA	NA
Thlaspi_arvense	+	KM361012	NA	NA
Thymus_praecox	+	KM361013	NA	NA
Thymus_pulegioides	+	KJ746270	NA	NA
Tilia_americana	+	HQ590302	NA	NA
Tilia_cordata	+	KP088884	NA	NA
Tipularia_discolor	+	AF074234	NA	NA
Tmesipteris_elongata	+	KJ569699	KJ569699	KJ569699
Tmesipteris_tannensis	+	EF469945	NA	NA
Torilis_arvensis	+	AM234827	NA	NA
Torilis_japonica	+	KM361016	NA	NA
Toxicoscordion_venenosum	+	KM242960	NA	NA
Trachycarpus_fortunei	+	AJ404752	NA	NA
Trachystemon_orientalis	+	KF158122	NA	NA
Tragopogon_pratensis	+	AY395563	NA	NA
Treubia_lacunosa	+	AY507428	NA	AY507468
Treubia_lacunosoides	-	AY507429	AY507515	AY507469
Tricoryne_elatior	+	JX903207	NA	NA
Trientalis_europaea	+	U96655	NA	NA
Trifolium_pratense	+	AY395564	KJ788290	KJ788290
Trifolium_repens	+	KM361020	NA	NA
Trifolium_subterraneum	+	NC_011828	NC_011828	NC_011828
Trillium_grandiflorum	+	D28164	NA	NA
Triplophyllum_vogelii	+	KF887210	NA	NA
Triraphis_mollis	+	JN681713	NA	NA
Trisetum_flavescens	+	AJ746276	NA	NA
Trisyngyne_balansae	+	L13344	NA	NA
Trisyngyne_codonandra	+	L13347	NA	NA
Triticum_aestivum	+	NC_002762	NC_002762	NC_002762
Tritomaria_exsectiformis	+	KC184766	KC184833	NA
Tritomaria_quinquedentata	+	AY700003	KF851855	KF851406
Trizeuxis_falcata	+	FJ534151	NA	NA
Tsuga_canadensis	+	JQ512625	NA	AY188220
Tsuga_mertensiana	+	AF145463	NA	AY188215
Typha_angustifolia	+	GQ436381	NA	X84147
Typha_latifolia	+	NC_013823	NC_013823	NC_013823
Ulmus_americana	+	AF500337	NA	NA
Vaccinium_myrtillus	+	KM361028	NA	NA
Vaccinium_oxycoccos	+	JN891672	NA	NA
Vaccinium_poasanum	+	JQ594910	NA	AY331865
Vaccinium_uliginosum	+	AF421107	NA	NA
Vaccinium_vitis_idaea	+	JN966057	NA	NA
Vachellia_erioloba	+	JX572192	NA	NA
Veratrum_oxysepalum	+	JN417478	NA	NA
Verbena_officinalis	+	Z37473	NA	NA
Verdoornia_succulenta	+	AY507430	AY507516	AY507470
Veronica_chamaedrys	+	KJ746275	NA	NA
Vicia_faba	+	KF042344	KF042344	KF042344
Vicia_hirsuta	+	HM850459	NA	NA
Vicia_sativa	+	NC_027155	NC_027155	NC_027155
Vicia_tetrasperma	+	HM850463	NA	NA
Vigna_unguiculata	+	NC_018051	NC_018051	NC_018051
Vincetoxicum_rossicum	+	KF539854	KF539854	KF539854
Vitis_vinifera	+	NC_007957	NC_007957	NC_007957
Wiesnerella_denudata	+	DQ286027	NA	DQ220701
Woollsia_pungens	+	U80425	NA	NA
Wullschlaegelia_aphylla	+	AY368436	NA	NA
Zamia_pumila	-	AY056557	NA	AY188209
Zea_mays	+	NC_001666	NC_001666	NC_001666
Zeuxine_agyokuana	+	HM141071	NA	NA
Zeuxine_strateumatica	+	KJ773994	NA	NA
Zingiber_officinale	+	KM213122	KM213122	KM213122
Zoysia_japonica	+	AM849385	NA	NA

Table S4 | Fossil calibration points used for divergence time estimates of land plants.

Crown group	Min divergence age	Max divergence age	Reference
Tracheophyta	416.0	454.0	(Cooper et al. 2012)
Spermatophyta	306.2	366.8	(Clarke et al. 2011)
Marchantiophyta	452.0	509.0	(Cooper et al. 2012)
Euphylllophyta	388.2	454.0	(Cooper et al. 2012)
Angiosperms	124.0	248	(Cooper et al. 2012)

Clarke, J. T., R. C. Warnock, and P. C. Donoghue. 2011. Establishing a time-scale for plant evolution. New Phytol 192:266-301.

Cooper, E. D., M. J. Henwood, and E. A. Brown. 2012. Are the liverworts really that old? Cretaceous origins and Cenozoic diversifications in Lepidoziaceae reflect a recurrent theme in liverwort evolution. Biol J Linn Soc 107:425-441.
